# The human IL-15 superagonist N-803 promotes migration of virus-specific CD8+ T and NK cells to B cell follicles but does not reverse latency in ART-suppressed, SHIV-infected macaques

**DOI:** 10.1371/journal.ppat.1008339

**Published:** 2020-03-12

**Authors:** Gabriela M. Webb, Jhomary Molden, Kathleen Busman-Sahay, Shaheed Abdulhaqq, Helen L. Wu, Whitney C. Weber, Katherine B. Bateman, Jason S. Reed, Mina Northrup, Nicholas Maier, Shiho Tanaka, Lina Gao, Brianna Davey, Benjamin L. Carpenter, Michael K. Axthelm, Jeffrey J. Stanton, Jeremy Smedley, Justin M. Greene, Jeffrey T. Safrit, Jacob D. Estes, Pamela J. Skinner, Jonah B. Sacha

**Affiliations:** 1 Vaccine & Gene Therapy Institute, Oregon Health & Science University, Beaverton, Oregon, United States of America; 2 Oregon National Primate Research Center, Oregon Health & Science University, Beaverton, Oregon, United States of America; 3 Department of Veterinary and Biomedical Sciences, University of Minnesota, St. Paul, Minnesota, United States of America; 4 ImmunityBio, Los Angeles, California, United States of America; 5 NantKWest, Los Angeles, California, United States of America; Emory University, UNITED STATES

## Abstract

Despite the success of antiretroviral therapy (ART) to halt viral replication and slow disease progression, this treatment is not curative and there remains an urgent need to develop approaches to clear the latent HIV reservoir. The human IL-15 superagonist N-803 (formerly ALT-803) is a promising anti-cancer biologic with potent immunostimulatory properties that has been extended into the field of HIV as a potential “shock and kill” therapeutic for HIV cure. However, the ability of N-803 to reactivate latent virus and modulate anti-viral immunity *in vivo* under the cover of ART remains undefined. Here, we show that in ART-suppressed, simian-human immunodeficiency virus (SHIV)_SF162P3_-infected rhesus macaques, subcutaneous administration of N-803 activates and mobilizes both NK cells and SHIV-specific CD8+ T cells from the peripheral blood to lymph node B cell follicles, a sanctuary site for latent virus that normally excludes such effector cells. We observed minimal activation of memory CD4+ T cells and no increase in viral RNA content in lymph node resident CD4+ T cells post N-803 administration. Accordingly, we found no difference in the number or magnitude of plasma viremia timepoints between treated and untreated animals during the N-803 administration period, and no difference in the size of the viral DNA cell-associated reservoir post N-803 treatment. These results substantiate N-803 as a potent immunotherapeutic candidate capable of activating and directing effector CD8+ T and NK cells to the B cell follicle during full ART suppression, and suggest N-803 must be paired with a *bona fide* latency reversing agent *in vivo* to facilitate immune-mediated modulation of the latent viral reservoir.

## Author summary

IL-15 regulates NK and memory T cell homeostasis and is therefore being explored for clinical immunotherapy of chronic diseases like cancer and HIV. To explore the applicability of the clinical grade IL-15 superagonist N-803 to HIV cure strategies we tested the impact of N-803 on host immunity and latent virus in SHIV-infected rhesus macaques. Our results suggest that N-803 beneficially modulates effector NK and CD8+ T cells by expanding the numbers of these cells and redistributing them to lymph node B cell follicles, a site known to harbor persistent latent virus during ART. However, our results further suggest that N-803 does not perturb the viral reservoir present in memory CD4+ T cells and that in order to fully unlock the immunotherapeutic potential of N-803 it must be paired with latency reversal agents.

## Introduction

The inability of antiretroviral therapy (ART) to clear the latent HIV reservoir, paired with the difficulties of life-long ART adherence, have shifted the focus of HIV research towards the development of therapies able to achieve a functional cure, namely ART-free remission from HIV. Proof-of-concept for HIV cure was provided by Timothy Brown, the “Berlin patient,” an HIV-positive individual who received a hematopoietic stem cell transplant from a CCR5^Δ32/Δ32^ donor as part of his treatment for leukemia [1]. Now over a decade after his transplant and discontinuation of ART, Timothy Brown shows no signs of HIV and continues to demonstrate that durable ART-free remission is an attainable goal [2]. Indeed, this remarkable result was recently confirmed by the report of the “London patient” [3]. HIV research has thus expanded its scope to include a wide variety of curative strategies including the most studied approach, “shock and kill” therapies [4]. These therapies function on the hypothesis that latency-reversing agents (LRAs) activate and flush out latent HIV for subsequent recognition and clearance by effector NK and CD8+ T cells. Several immune-based therapies are candidates to combat latent HIV and among these are the common γ-chain cytokines due to their capacity for robust T cell activation.

Common γ-chain cytokines have been previously explored as candidates to decrease HIV reservoir size in patients on ART. Interleukin-2 (IL-2) failed to decrease the latent HIV burden, while IL-7 modestly expanded the reservoir commensurate with its ability to expand CD4+ T cell numbers [5,6]. Furthermore, these cytokines did not disrupt viral latency. IL-15, however, a critical mediator of NK cell and T cell activation and proliferation, induces NK cell expansion and preferential proliferation of both CD4+ and CD8+ effector memory T cells in non-human primate models of HIV [7–9]. *In vitro*, IL-15 effectively reactivates HIV production in latently infected cells [10]. Therapeutic use of free IL-15, however, is precluded by its rapid plasma clearance and the high dose needed to achieve biological responses *in vivo*. Unlike IL-2 and IL-7, IL-15 is a part of a heterodimeric complex formed by a tight association between single-chain IL-15 and IL-15Rα [11]. Novel IL-15-based therapies such as N-803 (formerly ALT-803) and hetIL-15 [12] are now designed to mimic this association, thereby overcoming limitations of free IL-15. These compounds have already shown promise as potential immunotherapeutics in the context of HIV/SIV [12–14].

N-803 consists of a novel IL-15 mutant (N72D), containing an asparagine to aspartic acid mutation at position 72, which forms a stable heterodimeric complex with the alpha subunit of the IL-15 receptor (IL-15Rα). The N72D mutation in IL-15 is crucial for N-803 as it confers a 5-fold increase in its biological activity as compared to free IL-15 [15]. This complex is expressed as a structurally modified human IL-15N72D:IL15Rα:IgG1 Fc fusion protein that exhibits 25-fold higher biological activity and 35-fold longer serum half-life than soluble IL-15, which ultimately promotes stimulation of NK and memory T cells [16]. Accordingly, N-803 engenders potent anti-tumor NK and T cell immunity in small animal cancer models and is currently being tested as an immunotherapeutic for both solid and hematologic cancers in several different Phase I/II clinical trials (ClinicalTrials.gov; NCT01946789, NCT02099539). Moreover, N-803 is well-tolerated in both mice and cynomolgus macaques at doses as high as 0.1 mg/kg and importantly, does not induce global cytokine storm release of pro-inflammatory cytokines [17]. Given the promising results in the field of cancer immunology, N-803 has also been explored as a means to directly enhance anti-viral immune responses in chronic infections such as HIV [13,14]. For example, in a humanized mouse model of HIV, early administration of N-803 induced NK cell cytotoxicity and inhibited acute HIV replication [18]. More recently, N-803 demonstrated the remarkable ability to both reverse HIV latency and enhance CD8+ T cell recognition of HIV-infected cells in a primary *in vitro* cell culture model [10]. Furthermore, we have shown that a single dose of N-803 directs SIV-specific CD8+ T cells into B-cell follicles of chronically SIV-infected elite controller rhesus macaques [13]. These data support the hypothesis that N-803 could mediate both the “shock” and the “kill” in cure approaches for HIV, but as yet N-803 has not been tested in ART-suppressed macaques.

To explore this hypothesis, we evaluated the effect of the clinical grade IL-15 superagonist N-803 on the latent viral reservoir in SHIV-infected, ART-suppressed rhesus macaques. We demonstrate that biweekly, subcutaneous dosing of N-803 results in repeated proliferation of NK and memory CD8+ T cells. Within five days of N-803 administration, proliferating cells redistribute from blood to lymph nodes. Both NK cells and antigen-specific CD8+ T cells then gain access to B cell follicles within lymph nodes, a critical anatomical location that harbors latent virus. In contrast, we observed minimal activation and migration of CD4+ T cells. Although N-803 had no effect on reversing latency *in vivo* or on diminishing the viral cell-associated DNA reservoir, animals that received N-803 displayed a trend towards delayed viral rebound kinetics after ART interruption. Collectively, our data provide evidence that in the context of ART-suppression, N-803 can drive critical effector cells to sanctuary sites for viral replication and is a powerful tool for combinatorial HIV eradication strategies.

## Results

**Fig 1:**
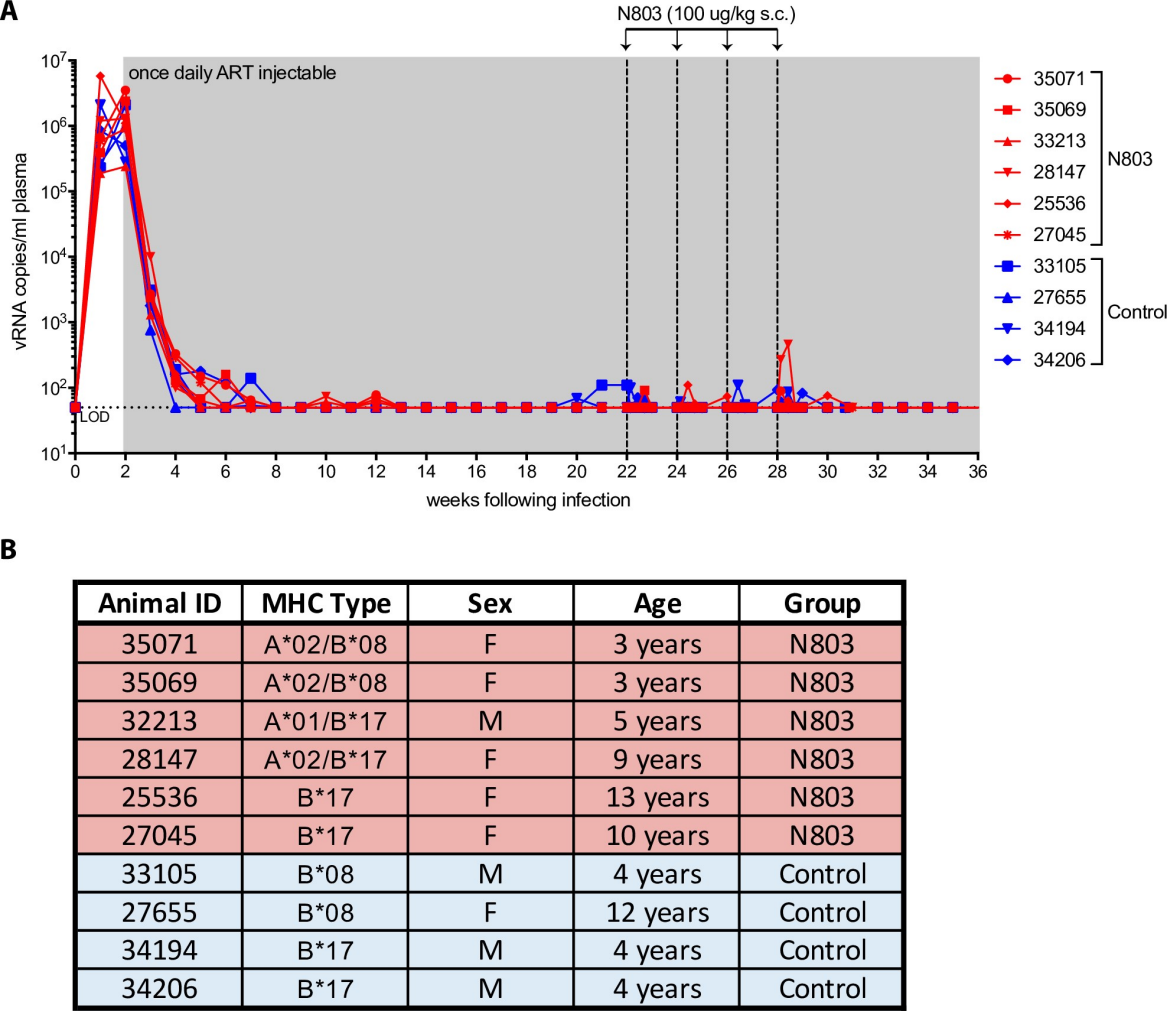
Plasma viral loads after infection with SHIVsf162p3 and before discontinuation of ART. We intravenously infected rhesus macaques (n = 10) with SHIVsf162p3 and initiated ART at week 2. The N-803 group (n = 6, red) received 4 subcutaneous infusions of N-803 (100 μg/kg) every other week, indicated by vertical dashed lines between weeks 22–28. The control group (n = 4, blue) was left untreated. Horizontal dotted line indicates limit of detection (LOD) at 50 RNA copies/mL. **(A)** Plasma viral loads shown prior to ART discontinuation and during N-803 treatment. **(B)** Table of research animal characteristics including animal identification number, MHC type, sex, age, and the group to which the animal was assigned.

Recent studies in mice have shown greater tissue biodistribution of N-803 to lymphoid organs when administered by subcutaneous administration as compared to intravenous administration [19]. In humans, subcutaneous versus intravenous administration resulted in a significantly longer serum half-life, decreased serum levels of pro-inflammatory cytokines, as well as sustained and significantly increased activation and proliferation of both NK cells and CD8+ T cells [20,21]. We have thus adjusted our dosing route for the current study from intravenous to subcutaneous. We infected 10 rhesus macaques (*Macaca mulatta*) intravenously with SHIV_SF162P3_ and initiated daily subcutaneous ART injections 14 days post-infection in order to establish the viral reservoir while allowing for the priming of SHIV-specific CD8+ T cell responses (**Fig 1A**) [22]. Because the MHC-I alleles Mamu-B*008:*01* (B*08) and Mamu-B*017:*01* (B*17) restrict particularly potent antiviral CD8+ T cell responses we selected and balanced animals positive for these alleles between the treatment and control groups to ensure the presence of high frequency anti-SHIV CD8+ T cells and to facilitate downstream *in situ* MHC-I tetramer staining (**Fig 1B)**. Although these MHC-I alleles are associated with spontaneous elite control of chronic phase viral replication, this effect does not manifest until six weeks post-infection at the earliest [23]. Therefore, no impact of these MHC-I molecules on viral replication was expected given that we began ART at two weeks post-infection. Following ART initiation, plasma viremia declined to undetectable levels in all animals by week eight. We continued daily ART for 20 weeks, wherein one group (n = 6) received four subcutaneous infusions of 100 μg/kg N-803 every other week over the course of eight weeks and the control group (n = 4) was left untreated. We continued to monitor viral loads throughout the course of N-803 treatments to look for episodes of virus reactivation (**Fig 1A**).

**Fig 2:**
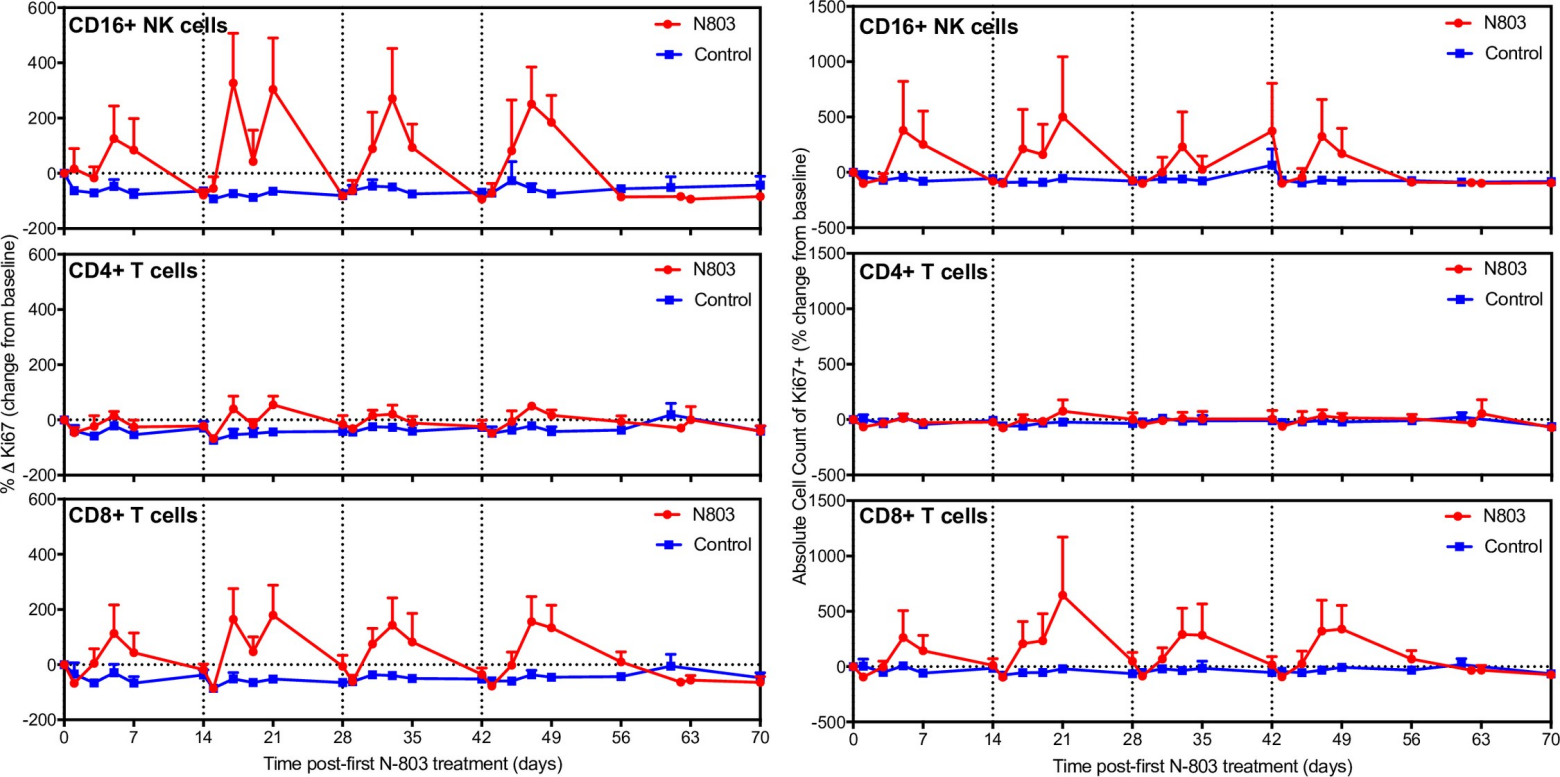
Whole blood analysis during *in vivo* administration of N-803. N-803 was subcutaneously administered every other week as indicated by the vertical dashed lines. Blood was collected at time 0 before the N-803 injection and at days 1, 3, 5, 7 after each injection of N-803. Induction of proliferation marker Ki67 on CD16+ NK cells, CD4+ and CD8+ T cells as percent of lymphocyte subset on the left, absolute cell counts on the right, both shown as a percent change from baseline. Absolute counts were calculated based on the percentage of the particular cell subset and the WBC count. Data shown are means (± SEM) of combined data from all animals within the designated group.

**Fig 3:**
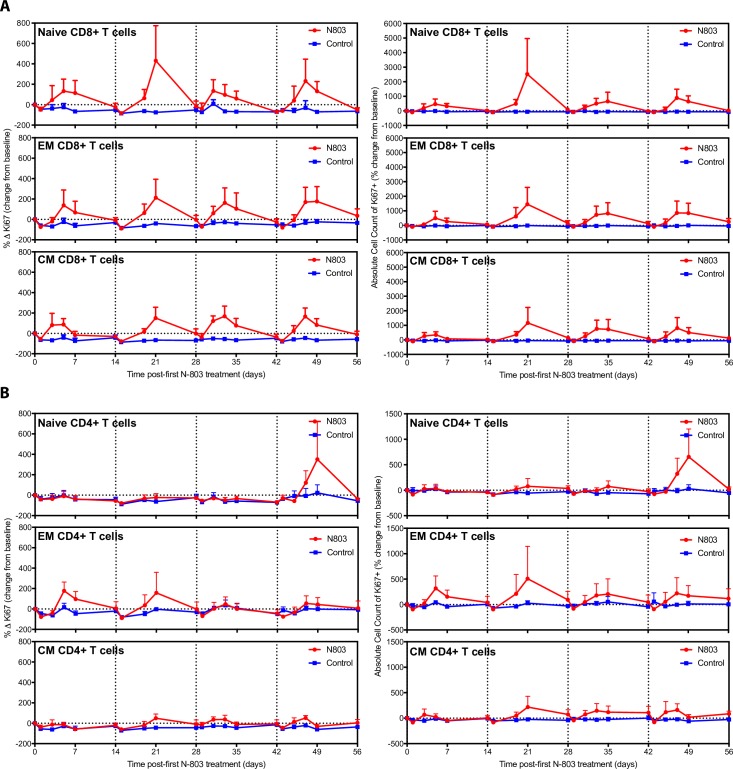
T cell memory analysis in blood during *in vivo* administration of N-803. **(A)** Induction of proliferation marker Ki67 on memory CD8+ T cells as percent of subset on the left, absolute cell counts on the right, both shown as a percent change from baseline. Absolute counts were calculated based on the percentage of the particular cell subset and the WBC count. **(B)** Induction of proliferation marker Ki67 on memory CD4+ T cells as percent of subset on the left, absolute cell counts on the right, both shown as a percent change from baseline. Absolute counts were calculated based on the percentage of the particular cell subset and the WBC count. Data shown are means (± SEM) of combined data from all animals within the designated group.

We, and others, have previously established that N-803 induces proliferation of immune effector cells, namely CD8+ T cells and NK cells, in SIV-infected RMs [13,14]. However, the effect is transient and resolves within 7–14 days post-administration. Repeated, weekly dosing of N-803 results in modulation of IL-15 receptor surface expression, including both IL-15/IL-2β (CD122) and the common γC (CD132) [14]. The decline in receptor surface expression likely reduces the responsiveness of T cells and NK cells to IL-15 over time. Hence, we administered N-803 every other week for eight weeks in order to allow for recovery of IL-15 responsiveness. Similar to previous reports on the effects of N-803 administered intravenously [13,14], the subcutaneous, bi-weekly dosing regimen used here resulted in a decrease in the absolute numbers of white blood cells, specifically CD8+ T cells and CD16+ NK cells, in blood one day post N-803 administration, likely due to trafficking into tissue [13,24], followed rapidly by an increase in the numbers of these cells in the blood (**S1A and S1B Fig)**. In contrast, we observed no increase in the number of CD4+ T cells in blood following subcutaneous N-803 administration. These repeated episodes of substantial expansion by NK and CD8+ T cells in the blood, and minimal CD4+ T cell activation, following subcutaneous N-803 were reflected in increases in proliferative activity as measured by expression of the proliferation marker Ki-67 in blood (**Fig 2**). We observed increased levels of Ki-67 expression in all memory subpopulations of CD8+ T cells, however, we only observed notable increases in the absolute numbers of effector and central memory CD8+ T cells in the blood after repeated N-803 treatments (**Figs 3A and** S2A). N-803 had minimal effects on memory subpopulations of CD4+ T cells, triggering only modest increases in Ki-67 expression in the effector memory population (**Figs 3B and** S2B). In line with minimal increased Ki-67 expression in memory CD4+ T cells, there was no associated increase in plasma viral loads of these animals during administration of N-803 (**Fig 1A**), suggesting that N-803 does not function as an LRA *in vivo*.

**Fig 4:**
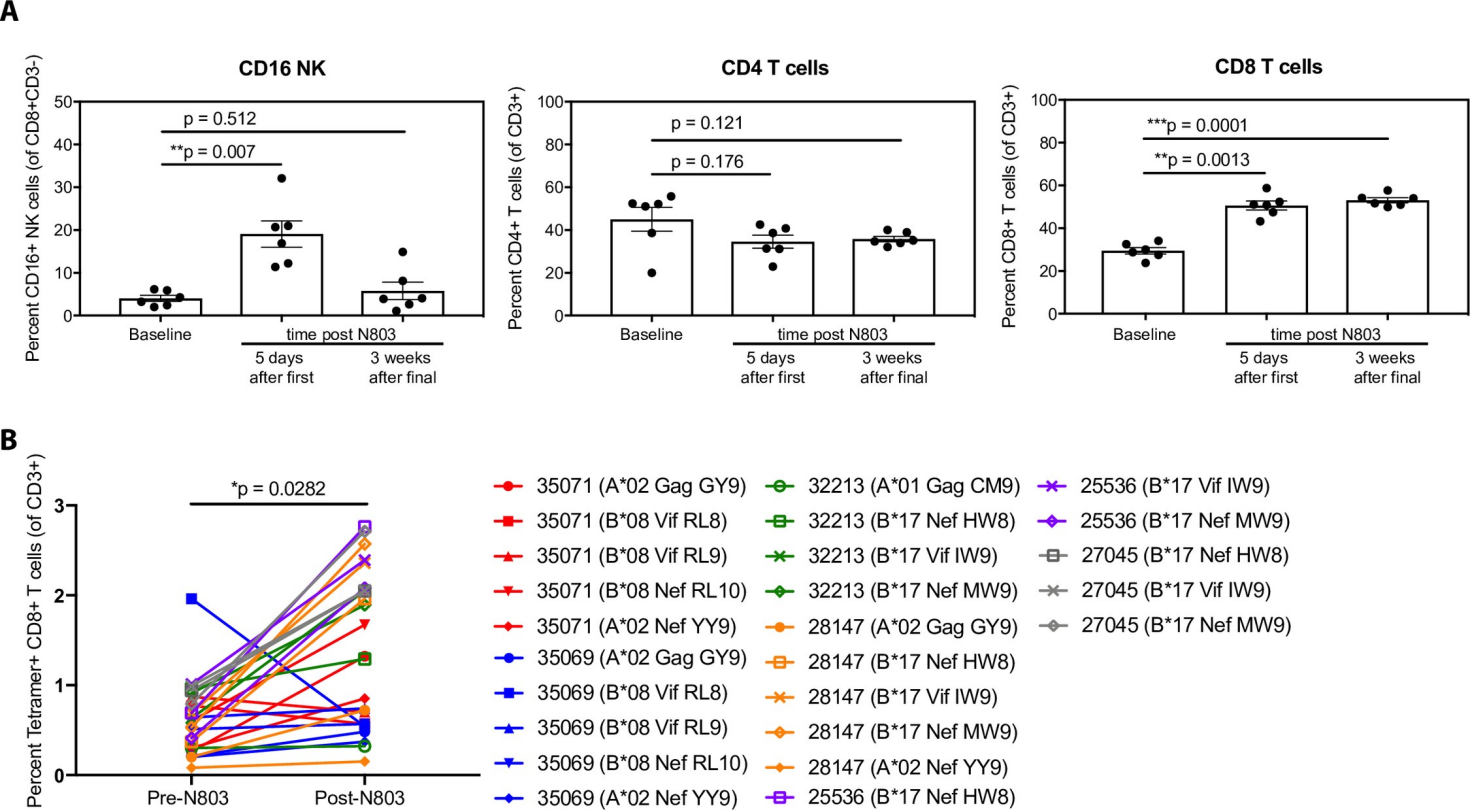
Subcutaneous administration of N-803 drives influx of CD16+ NK cells and SHIV-specific CD8+ T cells to lymph nodes. **(A)** The percentage of CD16+ NK cells, CD4+ T cells, and CD8+ T cells were determined in single-cell suspensions derived from axillary lymph nodes before, 5 days after the first injection of N-803 (100 μg/kg), and 3 weeks after the final dose of N-803. **(B)** Percent SHIV-specific CD8+ T cells as measured by MHC class I tetramer staining in lymph nodes. Animal IDs and MHC-I tetramer used are indicated (35071 = red, 35069 = blue, 32213 = green, 28147 = orange, 25536 = purple, 27045 = grey). P values were calculated using a paired t-test **(A)**, and a linear mixed effects model **(B)**. *, P<0.05; **, P<0.01; ***, P<0.001.

**Fig 5:**
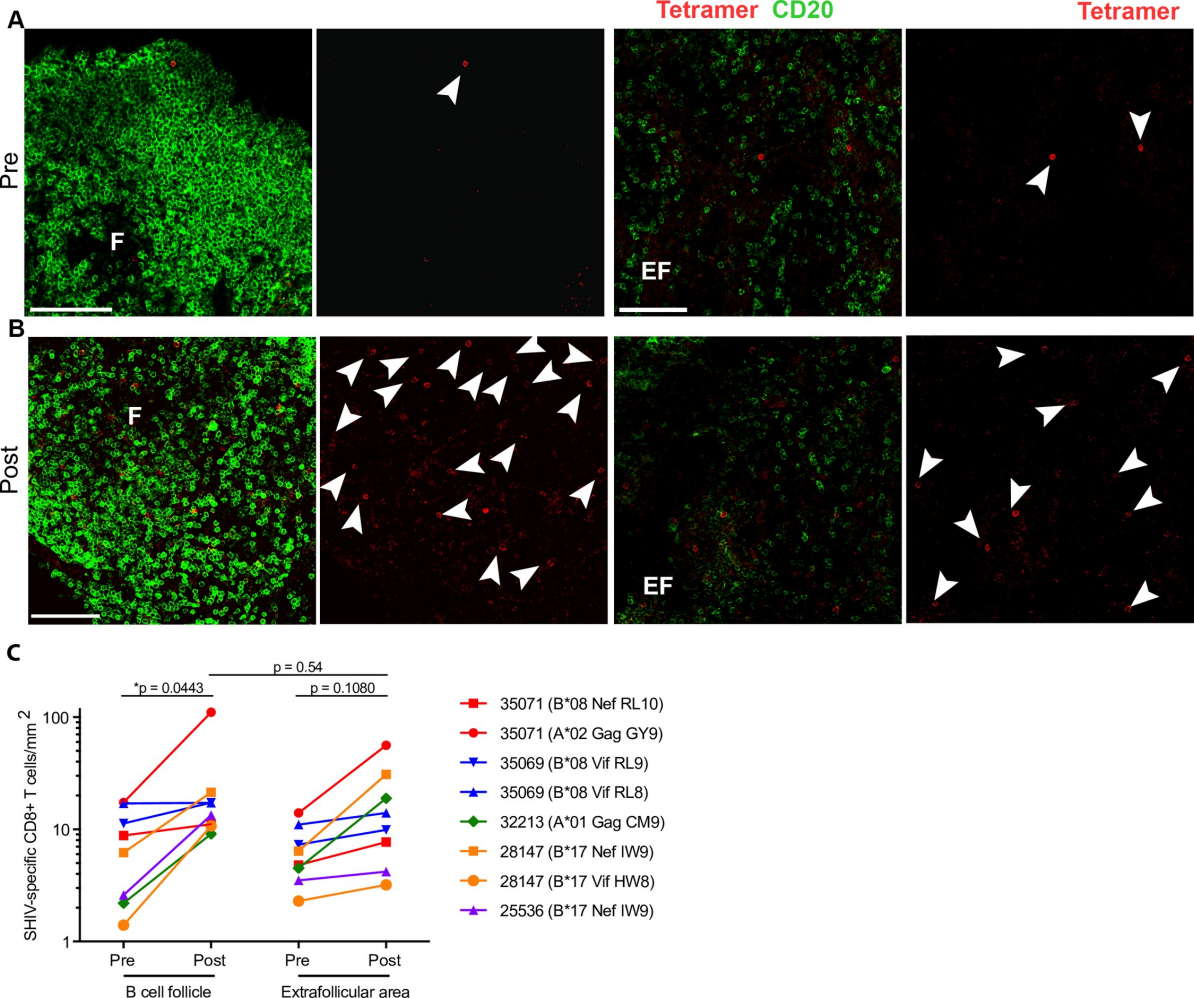
SHIV-specific CD8+ T cells increase in follicular and extrafollicular regions of lymph nodes after N-803 treatment in ART-suppressed macaques. Representative images showing Mamu-B*08 RL9 tetramer+ cells (red) and CD20+ cells (green) in lymph node sections from animal 35069, **(A)** before and **(B)** 5 days after N-803 treatment. CD20 staining is used to define B cell follicles (F) and extrafollicular (EF) regions. Tetramer-binding cell are indicated with white arrowheads. Scale bars indicate 200 μm (left panels) and 100 μm (right panels). **(C)** The numbers of tetramer+ CD8+ T cells per millimeter squared in B cell follicles and extrafollicular area before and 5 days after N-803 treatment. P values were calculated using a linear mixed effects model. *, P<0.05; **, P<0.01; ***, P<0.001.

**Fig 6:**
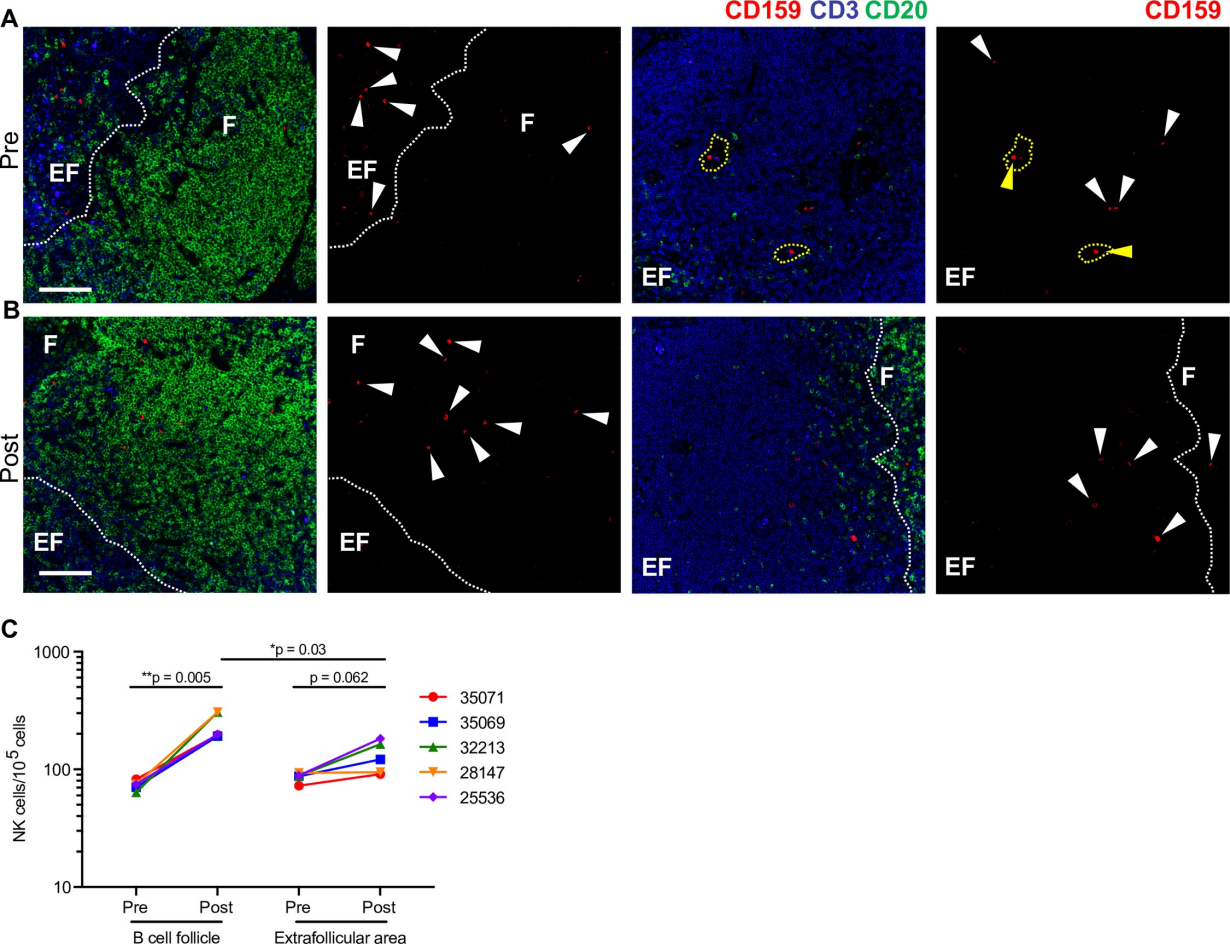
N-803 triggers NK cell migration from blood vessels and extrafollicular spaces into B cell zones of lymph nodes. Representative images from peripheral lymph nodes before (A) and 5 days after (B) N-803 treatment show a significant increase of NK cells (arrowheads) within B cell follicles (F) with only a modest increase in NK cells found in extrafollicular (EF) regions. Notably, many NK cells prior to N-803 treatment are found within blood vessels (yellow arrowheads), while most NK cells following N-803 treatment were found within the lymph node parenchyma (white arrowheads), specifically adjacent to or within B cell follicles. Scale bar is 100 μm. (C) Quantification of CD159a+CD3- cells within lymph nodes 5 days post-N-803 treatment demonstrated a significant increase in the number of NK cells found within B cell follicles. P values were calculated using a paired t-test. *, P<0.05; **, P<0.01; ***, P<0.001.

**Fig 7:**
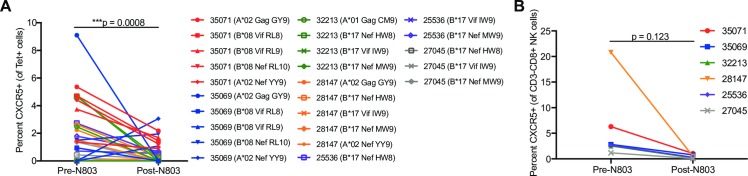
Decreased expression of CXCR5 on SHIV-specific CD8+ T cells and NK cells after N-803 in lymph nodes. **(A)** CXCR5 staining on SHIV-specific CD8+ T cells and **(B)** NK cells in peripheral lymph nodes before and after N-803 treatment. P values were calculated using a linear mixed effects model (**A**) and a paired t-test (**B**). *, P<0.05; **, P<0.01; ***, P<0.001.

In HIV patients with full ART suppression of viral replication, virus is latently-harbored within T follicular helper (T_FH_) cells, which remain a major source of persistent HIV [25,26]. An important focus of the HIV cure field has been to develop therapeutic strategies to direct potent effector cells into B cell follicles of lymph nodes to more effectively target and purge reactivated virus from latently HIV-infected T_FH_. We have previously established that N-803 administered intravenously in chronically SIV-infected elite controller RM induced SIV-specific CD8+ T cells to migrate from the blood to lymph nodes, where they gained access to B cell follicles [13]. To evaluate if subcutaneous administration of N-803 could also elicit trafficking of CD8+ T cells to lymph nodes in the antigen-deficient setting of ART suppression, we analyzed the frequency of CD16+ NK cells, as well as CD4+ and CD8+ T cells, within the lymph nodes five days after the first administration of N-803 (**Fig 4A**). Indeed, following a single subcutaneous dose of N-803, we observed significant increases in the numbers of both CD16+ NK cells and CD8+ T cells within lymph nodes (**Fig 4A**). In contrast to the transient influx of NK cells into the lymph node after N-803 administration, CD8+ T cell infiltration into lymph nodes was sustained up to 3 weeks after the final dose of N-803. In agreement with the minimal activation of CD4+ T cells in blood described above, there was no significant change in the number of CD4+ T cells found in the lymph nodes. Given the large, statistically significant increase in bulk CD8+ T cell numbers in lymph nodes, we next assessed whether virus-specific CD8+ T cells also accumulated in lymph nodes. We observed a statistically significant increase in the frequency of SHIV-specific CD8+ T cells in lymph nodes five days following N-803 treatment, as assessed by MHC-I tetramer staining of single cell suspensions of disaggregated lymph node (**Fig 4B**). This increase of SHIV-specific CD8+ T cells in lymph nodes was maintained for at least as long as three weeks after the final N-803 dose (**S3A Fig**). We next examined whether these virus-specific CD8+ T cells were able to gain access to B cell follicles of lymph nodes, an important anatomical site that harbors latent HIV and SIV. In order to assess this, we performed *in situ* SHIV-specific MHC-I tetramer staining of lymph node sections from five SHIV-infected RMs before and five days after subcutaneous administration of N-803. One macaque was excluded from all fixed tissue analysis due to high adipose content resulting in poor lymph node architecture. Prior to N-803 treatment, SHIV-specific CD8+ T cells primarily localized to the extrafollicular area within lymph nodes, and were largely excluded from the B cell follicles (**Fig 5A**). Five days after treatment with N-803, the frequency of SHIV-specific CD8+ T cells increased within the lymph nodes, achieving a statistically significant increase in the B cell follicle (**Fig 5B and 5C**). Given that we observed an increase in NK cell numbers in lymph nodes following N-803 treatment, we assessed the anatomical localization of effector NK cells within lymph nodes. Consistent with the results obtained for SHIV-specific CD8+ T cells, we observed a statistically significant increase of CD3-CD159a+ NK cells within B cell follicles five days after subcutaneous treatment with N-803 (**Fig 6**). Interestingly, we found a notable population of NK cells localized within blood vessels prior to N-803 treatment. Following N-803 administration, NK cells were primarily localized to the B cell follicle, and in some cases, NK cells were found within the extrafollicular area near, but not within blood vessels. This suggests that the increase in NK cells was due to extravasation instead of local proliferation. Finally, because of the increased NK and virus-specific CD8+ T cell trafficking into B cell follicles post subcutaneous N-803 administration, we evaluated the expression of CXCR5 on the surface of these cells using single cell suspensions of disaggregated lymph nodes taken before and five days post N-803 administration (**Fig 7**), as well as 3 weeks after the last dose of N-803 (**S3B and S3C Fig**). Despite finding higher numbers of both SHIV-specific CD8+ T cells and NK cells within B cell follicles following N-803 administration, we observed a statistically significant decrease in surface CXCR5 expression on lymph node-resident CD8+ T cells and a trend towards lower CXCR5 expression on NK cells (**Fig 7**). This paradoxical finding could be the result of temporal regulation of CXCR5 expression following N-803 activation or an indication that penetration of NK and CD8+ T cells into lymph node B cell follicles occurs via another undefined mechanism.

**Fig 8:**
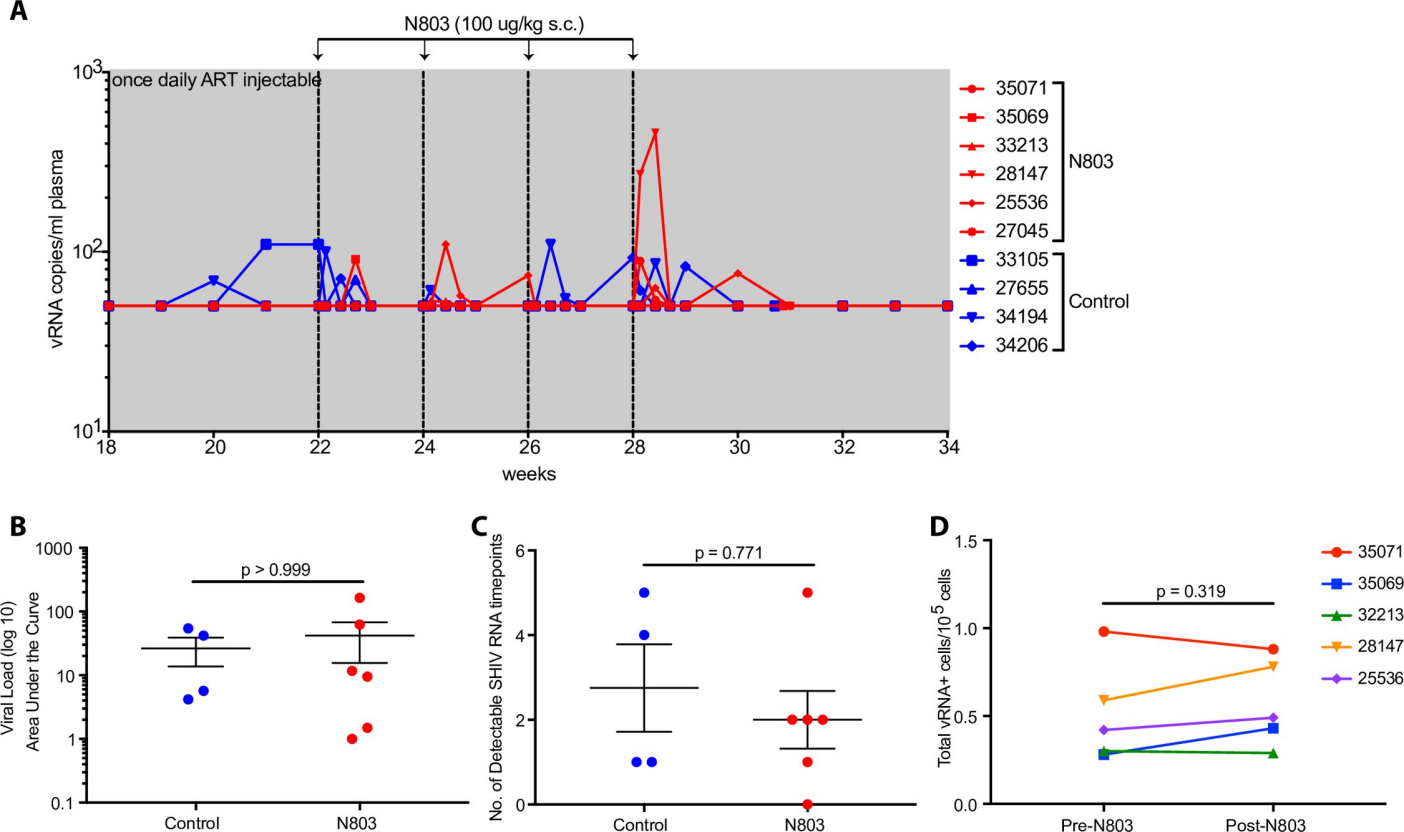
N-803 does not reverse latency *in vivo*. **(A)** Plasma viral loads shown during N-803 administration. N-803 was administered subcutaneously at a dose of 100 μg/kg every other week, indicated by the vertical dashed lines. Horizontal dotted line indicates limit of detection (LOD) at 50 RNA copies/mL. **(B)** Area under the curve analysis of viral loads between groups between weeks 22–35. **(C)** Number of detectable SHIV RNA timepoints between groups. **(D)** Quantification of RNAscope *in situ* hybridization in axillary lymph nodes before, 5 days after N-803 and 7 days after N-803. Shown as SHIV RNA+ cells per 100,000 cells. P values were calculated using Mann-Whitney test **(B, C)** and a paired t-test **(D)**. *, P<0.05; **, P<0.01; ***, P<0.001.

Recent data has shown that N-803 is able to reactivate latent virus from PBMC of ART-treated HIV-infected patients *ex vivo* [10]. However, there remains a lack of concordance between *in vitro* and *in vivo* HIV latency reversal activity. Hence, we sought to determine if N-803 could act as an LRA *in vivo* and induce episodes of virus reactivation when administered to fully ART-suppressed, SHIV-infected RMs. We measured plasma viral loads at 0, 1, 2, 3, and 7 days post administration during the course of N-803 treatment where we detected transient episodes of SHIV plasma RNA in RMs from both the untreated control group and the group that received subcutaneous N-803 (**Fig 8A**). We found no significant difference between the treatment and control groups in either the magnitude of the viral blips as measured by area under the curve (AUC) (**Fig 8B**), or in the number of detectable plasma SHIV RNA timepoints (**Fig 8C**). Additionally, we assessed for LRA activity by measuring the number of viral RNA+ cells present within the lymph node before and five days after N-803 treatment via quantitative RNAscope. In line with the lack of effect on plasma viral load, we observed no statistically significant change in the number of SHIV RNA+ cells in lymph node following N-803 treatment (**Fig 8D**). All animals received lymph node biopsies prior to and during the course of N-803 treatment, including the control group. The ‘blips’ in SHIV plasma RNA may be attributed to the additional stress of frequent tissue biopsies considering that the animals were fully ART-suppressed with undetectable plasma viremia for 11 weeks before N-803, and that only directly after the first biopsies were taken at week 19 did they have detectable virus in their plasma. Regardless, none of the results presented here support N-803 as an LRA *in vivo*. This is in agreement with a report published during the review of this manuscript describing that N-803 alone does not reserve SIV latency *in vivo* [27].

**Fig 9:**
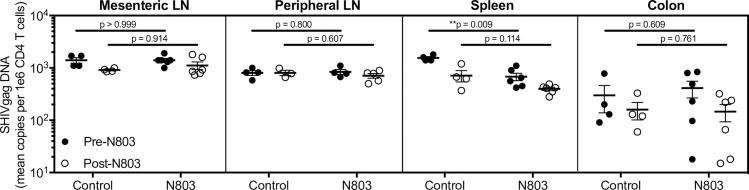
N-803 treatment does not perturb the viral reservoir. Cell-associated SHIV DNA from CD4-sorted, single-cell suspensions of tissues (mesenteric lymph nodes, peripheral lymph nodes, spleen, and colon) before N-803 and after all 4 injections of N-803. Data shown are means (± SEM). P values were calculated using a Mann-Whitney test. *, P<0.05; **, P<0.01; ***, P<0.001.

To assess if N-803 treatment impacted the size of the latent viral reservoir, we quantified cell-associated viral DNA in CD4+ T cells isolated from various tissues, including mesenteric and peripheral lymph nodes, spleen, and gastrointestinal mucosa, before and after all N-803 treatments. We observed similar levels of cell-associated viral DNA in the control and N-803 groups both before and following treatment, with the exception of the spleen which showed divergent levels of viral DNA at baseline, indicating that subcutaneous administration of N-803 by itself was not sufficient to decrease the viral reservoir (**Fig 9**). Importantly, unlike other common-γ chain cytokines such as IL-2 and IL-7, N-803 did not expand the viral reservoir [5,6].

**Fig 10:**
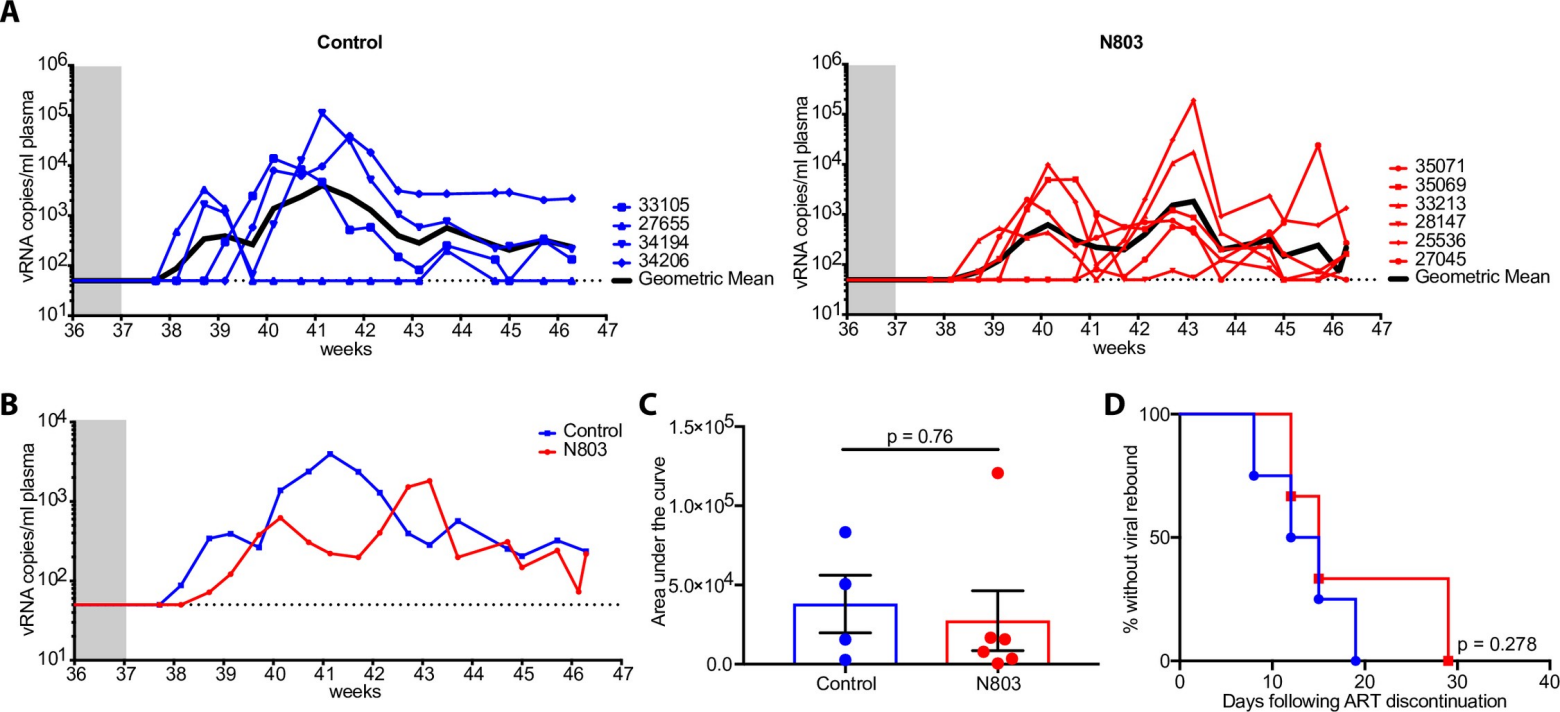
Animals that receive N-803 exhibit a slight delay in virus rebound after ART discontinuation. **(A)** Dynamics of SHIV viral load in plasma after ART release. Grey box indicates the end of ART regimen. Control group is shown on the left in blue and the N-803 group is shown on the right in red. Bold black line in each graph shows the geometric mean of each group. **(B)** Geometric means of both groups. **(C)** Area under the curve during the time of ART discontinuation to necropsy (week 37–46.5) **(D)** Kaplan-Meier curve showing the percent of animals with no viral rebound. Horizontal dotted line indicates limit of detection (LOD) at 62 RNA copies/mL. P values were calculated using a Mann-Whitney test **(C)**, and log-rank test **(D)**. *, P<0.05; **, P<0.01; ***, P<0.001.

Although we did observe blips in plasma viral loads prior to ART discontinuation, these events were not specific to N-803 administration as they occurred in both control and treatment groups. Additionally, CD4+ cell-associated viral DNA levels remained unchanged between the groups after N-803 treatment. We then wanted to determine if repeated N-803 treatment would affect the time to viral rebound after ART-interruption, defined as two detectable episodes above 62 copies/mL. At 37 weeks post-infection, nine weeks after the last dose of N-803 was administered, ART was discontinued and we monitored for any changes in the SHIV rebound kinetics (**Fig 10A**). We measured viral RNA in the plasma from all animals following ART cessation and all animals from both groups reached detectable levels of plasma viral RNA. Interestingly, the animals treated with N-803 displayed a trend towards delayed viral rebound kinetics as compared to the control animals (**Fig 10B–10D**). This trend could be due to the low animal numbers in the control group or to other unknown parameters (**Fig 10B–10D).** However, we took precautions to balance the animals in the groups such that the peak plasma viral loads and area AUC of plasma viremia prior to ART initiation were not statistically different between groups (**S4A and S4B Fig**). We also found no correlation between acute peak viral load and the time to rebound, although a correlation between peak acute phase viral load and peak viral load post ART cessation did exist (**S4C Fig**). Given that these animals had no significant differences in their virus reservoir size, this delay in rebound could be the result of an increase in effector cells, either or both NK cells and CD8+ T cells, elicited by N-803, thus providing the animals with more immune control, albeit transient, of rebounding virus following ART cessation.

## Discussion

Because of its ability to potently induce NK and T cell proliferation in cancer models (3,5,11,12), N-803, a novel IL-15 superagonist, is now a promising candidate for targeting and eradicating the HIV reservoir. In a humanized mouse model, treatment with N-803 led to suppression of acute HIV infection by increasing the antiviral activity of NK cells [18]. Additionally, *in vitro* evidence suggests N-803 may serve as a latency-reversal agent [10]. These data, together with our recent data showing that N-803 can drive SIV-specific CD8+ T cells to lymph nodes in SIV-infected RM [13], suggest that N-803 has the potential to flush virus out of latency and enhance killing of infected cells. Here we sought to determine if N-803 immunotherapy could indeed serve as both arms of the “shock and kill” strategy of HIV cure. In this study, we infected rhesus macaques with SHIV_SF162P3_ and initiated ART 14 days post-infection to sufficiently seed the viral reservoir [22,28]. While on ART, animals in the N-803 group received subcutaneous doses of N-803 every other week and we did not observe any notable differences in episodes of viral reactivation between the N-803 group and the control group. While our dosing regimen of N-803 elicited repeated and potent proliferation of NK cells and effector memory CD8+ T cells, as well as migration of these effectors to B cell follicles of lymph nodes, N-803 failed to impact the size of the viral reservoir. Interestingly, upon discontinuation of ART, animals that received N-803 experienced a slight delay in virus recrudescence. Our results reveal that although N-803 enhances trafficking of effector cells within B cell follicles of lymph nodes, it is not sufficient by itself to clear the latent virus reservoir, which further substantiates the need for combinatorial HIV cure approaches moving forward.

We previously defined that intravenous injection of N-803 triggers the influx of CD8+ T cells into lymph nodes of SIV-infected rhesus macaques [13]. Recent reports have compared the safety, tolerability, pharmacokinetics, immunologic events, and biodistribution between subcutaneous and intravenous routes of N-803 administration [21,29]. Although subcutaneous N-803 administration leads to 100-fold less serum concentrations compared to intravenous administration, it exhibits greater biodistribution to lymphoid organs. N-803 given via either route, however, induced comparable proliferation and activation of CD8+ T cells and NK cells. We have, therefore, adjusted our dosing route of N-803 from intravenous to subcutaneous, in order to more appropriately mirror the dosing route used in clinical trials currently testing N-803 in humans (NCT02523469, NCT03381586, NCT03054909, NCT03022825). Indeed, we found N-803 was still able to promote proliferation and activation of NK cells and T cells when administered subcutaneously. We also found that even with the development of anti-drug antibodies in four out of six of our treated animals, N-803 remained efficacious (**S5 and S6 Figs**). Interestingly, in contrast to our studies using intravenously administered N-803, we observed an influx of both CD8+ T cells and NK cells into lymph nodes with our subcutaneous administration of N-803. This could be due to the increased tissue biodistribution of N-803 observed via subcutaneous administration [29]. In addition to modifying our route of N-803 administration, we altered the timing of our dosing in order to achieve repeated proliferation of NK cells and T cells. We and others have shown that once a week dosing induces a burst in effector memory T cell and NK cell proliferation that then diminishes upon further injections, likely due to rapid recycling of the IL-15 receptors (CD122 and CD132) [8,14]. To mitigate this effect and allow for the recovery of IL-15 responsiveness, we adjusted our dosing schedule to biweekly N-803 and we were able to elicit potent proliferative responses in T cells and NK cells after each dose of N-803. Thus, with biweekly subcutaneous N-803 administration, we have achieved an effective dosing regimen for repeated proliferation of effector cells as well as trafficking of both NK cells and SHIV-specific CD8+ T cells from blood to B cell follicles.

The “shock-and-kill” strategy for HIV cure aims to combine a latency-reversing agent with effective immune-mediated killing of infected cells [30]. N-803 has recently been shown to induce HIV transcription from both primary cell models of latency and from *ex vivo* patient samples [10]. Therefore, we hypothesized that N-803 could act as both a latency-reversal agent and a potent immune-stimulant. To test this, we administered N-803 to rhesus macaques on suppressive ART and monitored the impact on plasma viremia. We found there to be no appreciable difference in the magnitude or number of transient plasma viremia events between animals that received N-803 versus animals that were left untreated. This was further confirmed via RNAscope of lymph node samples taken at five days post N-803 administration. Although a dose of 100 μg/kg N-803 given intravenously can reach maximum serum concentrations (C_max_) of 30 nM in cynomolgus macaques [17], recent findings demonstrate a 100-fold lower C_max_ of N-803 when administered subcutaneously compared to intravenously in mice [29]. Therefore, the lack of latency reversal activity could be due to the lower maximum serum concentration C_max_ of N-803 achieved with subcutaneous dosing. Given that animals in both groups experienced episodes of reactivation, it can also be argued that 20 weeks of ART suppression was not sufficient time to achieve complete viral suppression. Alternatively, a more sensitive viral load detection assay may be required to detect full suppression of less than 1 copy/mL, as is done in humans after long-term ART [31]. An important caveat to note is that we are using N-803, a human molecule, in a macaque model of SIV infection, which may exhibit different effects in macaques versus humans. We also failed to detect any changes in the viral reservoir within animals that received N-803. A limitation to our measurement, however, is that total SHIV gag DNA in CD4+ T cells includes replication defective proviruses, which vastly overestimates the size of the viral reservoir. While N-803 has been shown to potently activate and increase cytolytic potential of NK cells and CD8+ T cells *in vitro* [10,18,32], our previous *in vivo* studies indicate N-803 has no consistent impact on SIV-specific T cell responses in blood [13]. Intriguingly, upon ART discontinuation animals that received N-803 displayed slightly delayed rebound kinetics, despite evidence indicating N-803 had no effect on the viral reservoir. This may be due to the small number of animals studied, or the effect may be attributed to the increased frequency and tissue trafficking of immune effectors triggered by N-803, thus providing more immediate, although ultimately insufficient, immune control of recrudescent virus after ART interruption.

In conclusion, we have demonstrated that, in the context of ART suppression, N-803 is a valuable tool for combinatorial studies for HIV cure strategies. We have shown that subcutaneous dosing of N-803 enhances lymphocyte proliferation and activation and that biweekly dosing allows for the regeneration of IL-15-responsive cells. We have further established that subcutaneous N-803 delivery drives both NK cells and CD8+ T cells, including SHIV-specific CD8+ T cells to lymph nodes, granting them access to B cell follicles, a primary site of the HIV/SIV reservoir. However, we also demonstrated that subcutaneously administered N-803 does not appear to reverse latency *in vivo*, further supported by a recent report describing that N-803 alone does not reverse SIV latency *in vivo* [27]. Nevertheless, the semi-immune privileged cell follicle remains an obstacle to HIV cure [25,33] and N-803 can facilitate immune effector penetration of this barrier. Given this recent data, N-803 is likely not a viable option to mediate both the “shock” and the “kill” in eradication strategies, but instead can mediate “kill” and thus would be powerful in combination with a potent LRA. N-803 could also be combined with broadly neutralizing antibodies to promote antibody-dependent cell-mediated cytotoxicity by NK cells or with a therapeutic vaccine regimen to augment virus-specific CD8+ T cells. In sum, although N-803 itself does not appear to have any appreciable direct ability to reactivate latent virus, its immunotherapeutic benefits make N-803 an attractive candidate for combinatorial HIV eradication approaches.

## Methods

### Animals, reagents, and procedures

RM were infected intravenously with SHIV_SF162P3_, harvest 2 dated 9/12/2016 (100 TCID_50_), which was provided by Nancy Miller, National Institute of Allergy and Infectious Disease (NIAID), NIH. The TCID_50_ in PHA-activated rhesus PBMC is 1,758/ml and the TCID_50_ in TZMbl cells is 2.67 x 10^5^/ml. The p27 content of the stock is 182.79 ng/ml. ART treatment commenced two weeks post-infection and consisted of once daily subcutaneous injectable of tenofovir disoproxil fumarate (TDF; 5.1 mg/kg), emtricitabine (FTC; 40 mg/kg), and dolutegravir (DTG; 2.5 mg/kg) purchased from APIChem and formulated as described [28]. The IL-15 superagonist N-803 was generated by Altor Bioscience as previously described [34]. All N-803 injections were given as subcutaneous doses of 100 μg/kg. Axillary and inguinal lymph node biopsies, colon biopsies and laparoscopic collection of mesenteric lymph node and spleen were collected in accordance with the procedures outlined [35]. The Oregon Health & Science University Institutional Animal Care and Use Committee reviewed and approved all study protocols, which were in accordance with the U.S. Department of Health and Human Services’ Guide for the Care and Use of Laboratory Animals.

### Virus detection in plasma and tissue homogenates

Nucleic acid from plasma was purified using a Maxwell 16 instrument (Promega, Madison, WI) according to the manufacturer's protocol, using the LEV Viral Nucleic Acid Kit and the LEV Whole-Blood Nucleic Acid Kit, respectively. SHIV viral loads in plasma were determined by quantitative RT-PCR using the methods developed by Piatak *et al*. [36], except for a slightly modified master mix to increase sample input per reaction. SHIV viral loads in PBMC DNA were determined by quantitative PCR using Fast Advanced Mastermix on an Applied Biosystems QuantStudio 6 Flex instrument (Life Technologies, Carlsbad, CA). Reactions were performed with 2 μg nucleic acid input for 45 cycles using the FAST cycling protocol (95°C for 1 s, 60°C for 20 s) in a 30-μl reaction volume. Virus copy numbers were estimated by comparison to a linearized pBSII-SIV*gag* standard curve and calculated per cell equivalent using the input nucleic acid mass and by assuming a DNA content of 6.5 μg per million cells. Primers and probe used for plasma and PBMC assays were those described by Piatak *et al*. [36]: SGAG21 forward (GTCTGCGTCATPTGGTGCATTC), SGAG22 reverse (CACTAGKTGTCTCTGCACTATPTGTTTTG), and pSGAG23 (5′-(FAM)-CTTCPTCAGTKTGTTTCACTTTCTCTTCTGCG-(BHQ1)-3′).

For viral DNA reservoir detection in tissues, a recently developed ultrasensitive nested quantitative PCR [37] targeting a highly conserved region in SIV and SHIV *gag* was used. Primers used for DNA pre-amplification were SIVnestF01 (GATTTGGATTAGCAGAAAGCCTGTTG) and SIVnestR01 (GTTGGTCTACTTGTTTTTGGCATAGTTTC). Primers used for quantitative PCR were SGAG21 forward, SGAG22 reverse, and pSGAG23 as described above. Briefly, samples were heated at 95°C for 5 min and then put on ice. Each sample was assayed in 12 replicates (5 μg each), with two of the reactions including a spike of 10 or 20 copies of DNA or RNA, respectively, containing the SIV *gag* target sequence in order to assess PCR reaction efficiency. None of the tested DNA samples showed significant amplification inhibition, which was defined as a 5-cycle amplification delay as compared to the amplification kinetics of reactions containing solely 10 copies of standard. First-round amplification involved 12 cycles (95°C for 30 s and 60°C for 1 min) in 50-μl reactions. Then, 5 μl of each pre-amplified replicate was assayed by quantitative PCR using Fast Advanced Mastermix in a 30-μl reaction volume in the QuantStudio 6 Flex instrument. Reactions were performed for 45 cycles using the FAST cycling protocol. Virus copy numbers were derived from the frequency of positive replicates using the Poisson distribution and calculated as copies per μg of DNA. Staff members performing the DNA assays were blinded to the plasma and tissue samples that were being tested for virus. The limit of detection was 50 copies/mL, until the time of ART release (week 37 on) where the limit of detection was 62 copies/mL.

### Blood and tissue processing

Whole blood was collected into EDTA-treated tubes (BD Biosciences, San Jose, Ca, USA). Blood was assessed for complete blood counts using an ABX Pentra 60 C+ (Horiba, Irvine, CA, USA). Colon was finely diced and placed in a 50 ml conical containing 25 ml RPMI 1640, supplemented with 3% FCS (R3; Hyclone Laboratories, Logan, UT, USA). DTT was added at a final concentration of 200 μM, and tissues were shaken at 225 rpm for 15 min at room temperature. Tissues were allowed to settle, and the R3 with DTT was aspirated and replaced with R3 containing 5 mM EDTA. Tissues were shaken at 225 rpm for 30 min at 37°C, and the cell-containing supernatant was collected and passed through a cell strainer. R3 containing EDTA was added again, tissues shaken, and cells collected. Tissues were washed three times in 1X HBSS to remove excess EDTA and then were suspended in R3 containing 0.2 mg/ml collagenase (Sigma-Aldrich, St. Louis, MO, USA) and 0.2 mg/ml DNase I (Roche, Indianapolis, IN, USA). Tissues were shaken at 225 rpm for 1 hour at 37°C, and the cell-containing supernatant was collected and passed through a metal strainer. Cell fractions collected from the EDTA and collagenase digestion steps were combined (total tissue) and resuspended in 70% isotonic Percoll (GE Healthcare, Buckinghamshire, UK). The cells were then underlayed in 37% Percoll gradient and spun at 500 *g* with the brake off. Mononuclear cells from the lower interface were collected and washed in RPMI 1640 containing 10% FCS (R10). Lymph node and spleen were diced with scalpels and then forced through a 70-μm cell strainer. The strainer was rinsed repeatedly with R10 to obtain a single-cell suspension. Immune cell phenotyping was conducted on whole blood samples that were washed twice in 1X PBS and then surface-stained for 30 minutes at room temperature. CD4+ and CD8+ T cell memory populations were determined via CD28 and CD95 staining. Samples were then incubated in 1 ml FACS lyse for 8 minutes, spun at 830 *g* for 4 minutes, and washed three times in 1X PBS, supplemented with 10% FCS (FACS buffer). For intracellular Ki67, granzyme B and perforin assessment, fixed cells were washed twice with FACS buffer and incubated for 10 minutes with 1X FACS Perm (1X FACS lyse with 0.05% Tween20). Cells were then washed three times with FACS buffer and stained with intracellular Ki67, granzyme B, and perforin at room temperature for 45 mins. Cells were then washed once with FACS buffer and then run on an LSR II (Becton Dickinson, Franklin Lakes, NJ, USA). Flow cytometric data were analyzed using FlowJo, version 10 (TreeStar Ashland, OR, USA).

### *In situ* tetramer staining combined with immunohistochemistry

*In situ* tetramer staining combined with immunohistochemistry was performed on fresh lymph tissue specimens shipped overnight, sectioned with a compresstome and stained essentially as previously described [33]. Biotinylated peptide-loaded MHC-class I monomers for Mamu-A1*001:01 Gag_181-189_CM9, Mamu-A1*002:01 Gag_71-79_GY9, Mamu-B*08:01 Vif_172-179_RL8, Mamu-B*08:01 Vif_123-131_RL9, Mamu-B*08:01 Nef_137-146_RL10, Mamu-B*17:01 Nef_165-173_IW9, Mamu-B*17:01 Vif_66-73_HW8 (National Institute of Health Tetramer Core Facility, Emory University, Atlanta GA) were converted to FITC-labeled MHC-class I tetramers. The MHC-tetramers used to stain antigen specific T cells in tissues were limited by the amount of tissue sections obtained from lymph node biopsies, and by the FITC-labeled MHC-tetramers we had available for *in situ* staining. We thus focused our studies on MHC tetramers that recognize predicted immunodominant T cell responses. Fresh lymph node sections were incubated with MHC-class I tetramers (0.5 μg/ml) and rat-anti-human CD8 antibody (2 μg/mL, clone YTC182.20, Acris). For secondary incubations, sections were incubated with 1) rabbit-anti-FITC Abs (0.5 μg/mL, BioDesign, Saco, ME) and mouse-anti-human CD20 Abs (0.19 μg/mL, clone L26, Novocastra), or 2) mouse-anti-human CD20 Abs (0.19 μg/mL, clone L26, Novocastra) and rat-anti-human CD3 Abs (2 μg/mL, clone CD3-12, BioRad). For the tertiary incubations, all sections were incubated with Cy3-conjugated goat-anti-rabbit Abs (0.3 μg/mL, Jackson ImmunoResearch Laboratories), Alexa 488-conjugated goat-anti-mouse Abs (0.75 μg/mL, Molecular probes), and Cy5-conjugated goat anti-rat Abs (0.3 μg/mL, Jackson ImmunoResearch Laboratories). Sections were imaged using a Leica DM6000 confocal microscope. Montage images of multiple 512 × 512 pixels were created and used for analysis. Confocal z-series were collected in a step size of 3 μm.

### Quantification of SHIV-specific CD8 T cells *in situ*

Images were opened and analyzed in LAS X (Leica confocal) software directly. Follicular areas were identified morphologically as clusters of brightly stained, closely aggregated CD20^+^ cells. Follicular and extrafollicular areas were delineated and measured using LAS X software. Areas that showed loosely aggregated B cells that were ambiguous as to whether the area was a follicle were not included. To prevent bias, the red tetramer channel was turned off when follicular and extrafollicular areas were delineated. Cell counts were done on single z-scans. An average of 16 tetramer+ cells (range, 1–101) in follicular regions and 23 (range,0–97) in extrafollicular regions in each animal were analyzed. An average of 1.766 mm^2^ (0.45–5.03 mm^2^) was evaluated for each lymph node.

### Fluorescence microscopy for NK cell analysis

Fluorescence microscopy was performed on formaldehyde fixed, paraffin-embedded (FFPE) tissue sections (5 μm) according to our previously published protocol [38] with the following minor modifications: antigen retrieval was performed with citrate pH 6 without protease treatment and antigen stripping was performed by incubating slides in heated citraconic anhydride antigen retrieval buffer (95°-99°C) for 10 min. The antibodies used were anti-CD159a (Sigma-Aldrich; HPA004471), anti-CD3 (ThermoFisher; clone SP7), and CD20 (Biocare; clone L26). Detection was performed sequentially with polymer horseradish peroxidase (HRP)-conjugated systems (GBI Labs) coupled with tyramide-conjugated Alexa Fluors (ThermoFisher). Slides were counterstained with DAPI, cover slipped using ProLong Gold Antifade Mountant (ThermoFisher; P36930), and scanned on an Axio Scan.z1 at 20x (Zeiss). Blood vessels were defined as morphologically-distinct channels containing RBCs, which are dual autofluorescent in the 488 and 594 channels. In the images provided, the RBCs within the yellow dotted regions appear to be a bright blue, due to the pseudocoloring of the image and the intensity of the other colors in the image which make them appear single positive.

### SHIV RNA *in situ* hybridization

RNAscope was performed on FFPE tissue sections (5μm) according to our previously published protocol [39] with the following minor modifications: heat-induced epitope retrieval was performed by boiling slides in 1x target retrieval (322000; ACD) for 30 min, followed by incubation at 40°C with a 1:10 dilution of protease III (322337; ACD) in 1x PBS for 20 min. Slides were incubated with the target probe SIVmac239 (312811; ACD) for 2 hours at 40°C. Amplification was performed with RNAscope 2.5 HD Detection kits (322360; ACD) according to manufacturer’s instructions, with 0.5X wash buffer (310091; ACD) used through amplification step 4 and TBS-T used from amplification step 5 until the end of the assay. The resulting signal was detected with Warp Red chromogen (WR806M; Biocare Medical). Slides were counterstained with CAT hematoxylin (CATHE-GL; Biocare Medical), mounted with Clearmount (17885–15; EMS) until dry, coverslipped using Permount (SP15-100; Fisher Scientific), and scanned at 40x magnification on an Aperio AT2 (Leica Biosystems).

### Quantitative image analysis

Images were quantitatively analyzed using Halo software (v2.3.2089.27; Indica Labs). For NK cell quantification, the random forest classifier was used to define anatomical regions based on CD3 and CD20 staining and embedded within the Cytonuclear FL v1.4 analysis module. NK cells were defined as CD159^+^CD3^-^ cells and quantified relative to the total cells within each specific anatomical region. NK cells present within blood vessels were excluded from quantification. For RNAscope analysis, the ISH v2.2 module settings were set based on concomitantly assayed, early chronically infected SIV^+^ control slides to determine the vRNA minimum signal size necessary to exclude detection of the smaller single vRNA and/or vDNA molecules. Single copy size was set at 0.49 μm^2^ and the threshold for analysis was set at 0.85 μm^2^. Total cell counts were determined with the Cytonuclear v1.6 module to increase accuracy. In all quantifications, manual curation was performed on each sample to correct for false positives/false negatives.

### N-803 Antidrug Antibody (ADA) Assay

MSD 96-well SECTOR plate (Meso Scale Diagnostic, LLC) was coated by adding 50 μL per well of 2.5 μg/mL of N-803 in PBS and incubated overnight at 4°C. The plate was washed 3x with PBS-T and blocked with 150 μL per well of blocking solution (5% BSA in PBS) for 1 hour with shaking at 700 rpm at room temperature. To make the standard curve, a 4-fold dilution series of mouse anti-IL15 (R&D systems) was prepared using 10% normal cynomolgus monkey serum (Abcam) in assay buffer (1% BSA in PBS); the concentrations of the standards were 400, 100, 25, 6.25, 1.562, 0.391, 0.098, and 0 ng/mL. All serum samples were diluted 1:10 in assay buffer. After a 1 hour incubation, the blocking solution was removed from the plate. 50 μL of 0.0625 μg/mL SULFO-tag conjugated N-803 in assay buffer and 25 μL of standards and diluted serum samples were added to each well and incubated for 2 hours with shaking at 700 rpm at room temperature. The plate was washed 3x with PBS-T and 150 μL of 2X Read Buffer T (Meso Scale Diagnostic, LLC) was added to each well before reading on the MSD reader SECTOR S 600 (Meso Scale Diagnostic, LLC).

### Ethics statement

All rhesus macaques (RMs) (*Macaca mulatta*) in this study were managed according to the ONPRC animal husbandry program, which aims at providing consistent and excellent care to nonhuman primates. This program is based on the laws, regulations, and guidelines set forth by the United States Department of Agriculture (e.g., the Animal Welfare Act and its regulations, and the Animal Care Policy Manual), Institute for Laboratory Animal Research (e.g., Guide for the Care and Use of Laboratory Animals, 8th edition), Public Health Service, National Research Council, Centers for Disease Control, the Weatherall Report titled “The use of nonhuman primates in research”, and the Association for Assessment and Accreditation of Laboratory Animal Care (AAALAC) International. The nutritional plan utilized by the ONPRC is based on National Research Council recommendations and supplemented with a variety of fruits, vegetables, and other edible objects as part of the environmental enrichment program established by the Behavioral Management Unit. Paired/grouped animals exhibiting incompatible behaviors were reported to the Behavioral Management staff and managed accordingly. All efforts were made to minimize suffering through the use of minimally invasive procedures, anesthetics, and analgesics when appropriate. Animals were painlessly euthanized with sodium pentobarbital and euthanasia was assured by exsanguination and bilateral pneumothorax, consistent with the recommendations of the American Veterinary Medical Guidelines on Euthanasia (2013).

### Statistics

Data from whole blood analysis is displayed as a percent change from baseline calculated as such: ((value-baseline)/baseline) x 100 = percent change. Log-rank test was used for analysis of Kaplan-Meier curve comparisons. Analyses between groups was performed using Mann-Whitney U test. To account for within animal correlation in tetramer analysis, a linear mixed effects model was used. For each outcome, the final sensible model was selected using AIC. Fixed effects considered include time and tetramer specificity. Random effects components were selected between random intercept only and random intercept and random slope for time. Visual model diagnostics were performed to detect severe violations to the assumptions of linear mixed effect model. Analysis was performed using SAS9.4. Log-rank test was used for Kaplan-Meier curve analysis. For correlations, linear regression with Pearson’s correlation was used. Paired Student’s t test was used for all other analyses. Statistical analyses were conducted using GraphPad Prism version 6.0 (GraphPad Software, La Jolla, California, USA). Statistical significance of the findings was set at a *p*-value of less than 0.05.

## Supporting information

S1 Fig Whole blood analysis during *in vivo* administration of N-803. N-803 was subcutaneously administered every other week as indicated by the vertical dashed lines. Blood was collected at time 0 before the N-803 injection and at days 1, 3, 5, 7 after each injection of N-803. **(A)** White blood count (WBC), lymphocytes, monocytes, and neutrophils were analyzed from blood. **(B)** CD16+ NK cells, CD4+ T cells, CD8+ T cells were analyzed from blood and shown as a percent of CD45+ cells, absolute cell counts on the right, both shown as a percent change from baseline. Absolute counts were calculated based on the percentage of the particular cell subset and the WBC count. Data shown are means (± SEM) of combined data from all animals within the designated group.
(TIF)

S2 Fig Whole blood analysis during *in vivo* administration of N-803. N-803 was subcutaneously administered every other week as indicated by the vertical dashed lines. Blood was collected at time 0 before the N-803 injection and at days 1, 3, 5, 7 after each injection of N-803. Memory subpopulations (naïve, effector memory, central memory) of **(A)** CD8+ T cells and **(B)** CD4+ T cells. On the left is the percent of CD8+ or CD4+ T cells and absolute cell counts are on the right, both shown as a percent change from baseline. Absolute counts were calculated based on the percentage of the particular cell subset and the WBC count. Data shown are means (± SEM) of combined data from all animals within the designated group.
(TIF)

S3 Fig Dynamics of SHIV-specific CD8+ T cells and CXCR5 in the lymph nodes during and after N-803 administration. **(A)** Percent SHIV-specific CD8+ T cells as measured by MHC class I tetramer staining in lymph nodes prior to N-803, 5 days after N-803, and 3 weeks after the final N-803 administration. **(B)** CXCR5 staining on SHIV-specific CD8+ T cells in lymph nodes prior to N-803, 5 days after N-803, and 3 weeks after the final N-803 administration. **(C)** CXCR5 staining on NK cells in lymph nodes prior to N-803, 5 days after N-803, and 3 weeks after the final N-803 administration. P values were calculated using a paired t-test. *, P<0.05; **, P<0.01; ***, P<0.001.
(TIF)

S4 Fig Viral load analysis and correlations of viral rebound. **(A)** Peak plasma viral loads and **(B)** area under the curve of viral loads prior to ART discontinuation. **(C)** Correlation of peak viral load post-ART release with pre-ART peak viral load. **(D)** Correlation of the time to the first detectable viral RNA in plasma after ART release with pre-ART peak viral load. Data shown are means (± SEM). P values were calculated using a Mann-Whitney test **(A, B)**, and linear regression with Pearson’s correlation **(C, D)**. *, P<0.05; **, P<0.01; ***, P<0.001.
(TIF)

S5 Fig Anti-drug antibody and CD16+ NK cell count during the course of N-803 administration. Anti-drug antibody development in each animal that received N-803 and the absolute cell count of CD16+ NK cells. Vertical dashed lines indicate times of N-803 administration.
(TIF)

S6 Fig Anti-drug antibody and CD8+ T cell count during the course of N-803 administration. Anti-drug antibody development in each animal that received N-803 and the absolute cell count of CD8+ T cells. Vertical dashed lines indicate times of N-803 administration.
(TIF)
