# Supplementary figures and images for: The human IL-15 superagonist N-803 promotes migration of virus-specific CD8+ T and NK cells to B cell follicles but does not reverse latency in ART-suppressed, SHIV-infected macaques
Source: PLoS Pathog. 2020 Mar 12;16(3):e1008339. doi: 10.1371/journal.ppat.1008339 (PMC7093032; doi:10.1371/journal.ppat.1008339)

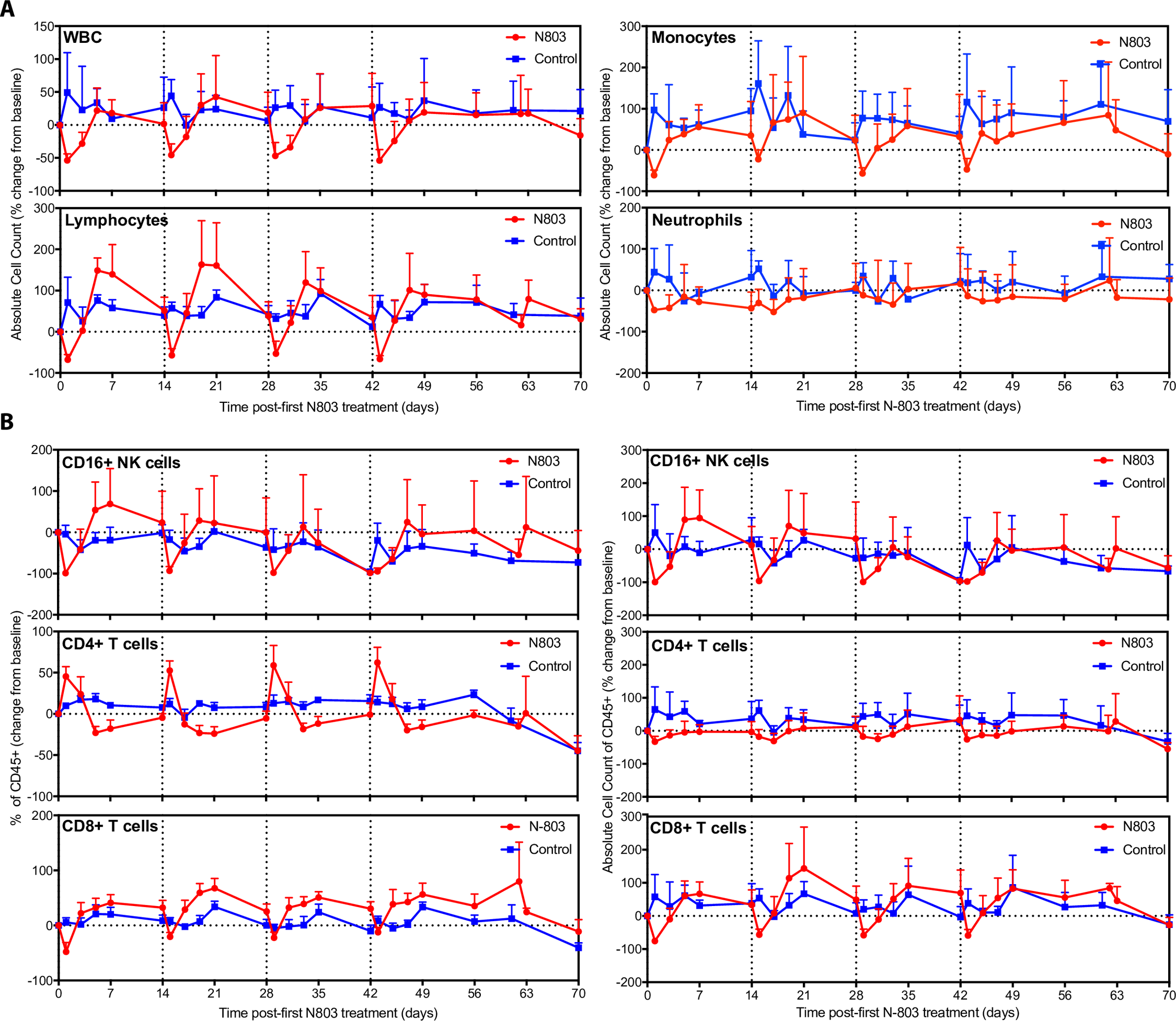

Supplement: S1 Fig — N-803 was subcutaneously administered every other week as indicated by the vertical dashed lines. Blood was collected at time 0 before the N-803 injection and at days 1, 3, 5, 7 after each injection of N-803. (A) White blood count (WBC), lymphocytes, monocytes, and neutrophils were analyzed from blood. (B) CD16+ NK cells, CD4+ T cells, CD8+ T cells were analyzed from blood and shown as a percent of CD45+ cells, absolute cell counts on the right, both shown as a percent change from baseline. Absolute counts were calculated based on the percentage of the particular cell subset and the WBC count. Data shown are means (± SEM) of combined data from all animals within the designated group. (TIF) [file ppat.1008339.s001.tif]

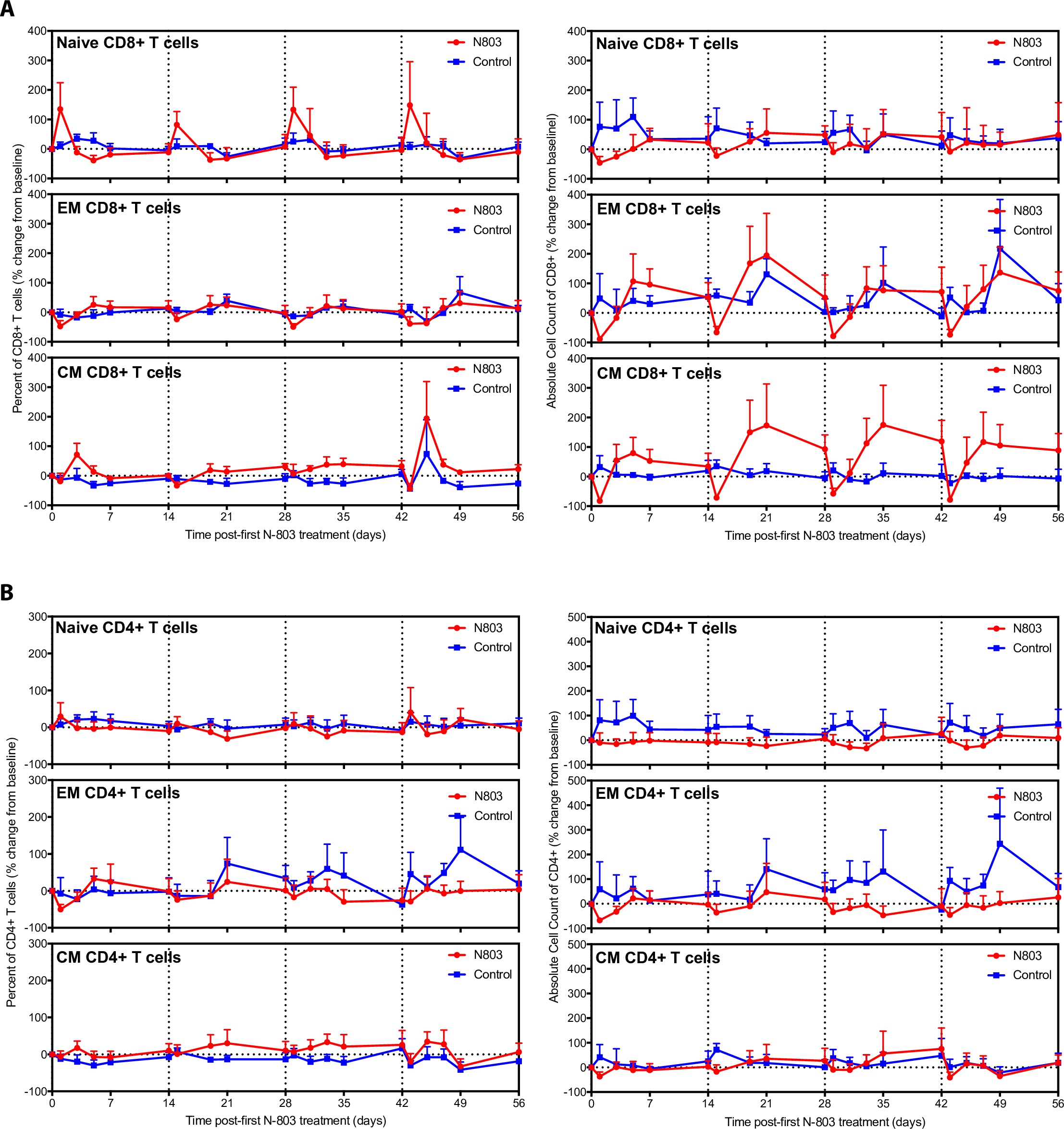

Supplement: S2 Fig — N-803 was subcutaneously administered every other week as indicated by the vertical dashed lines. Blood was collected at time 0 before the N-803 injection and at days 1, 3, 5, 7 after each injection of N-803. Memory subpopulations (naïve, effector memory, central memory) of (A) CD8+ T cells and (B) CD4+ T cells. On the left is the percent of CD8+ or CD4+ T cells and absolute cell counts are on the right, both shown as a percent change from baseline. Absolute counts were calculated based on the percentage of the particular cell subset and the WBC count. Data shown are means (± SEM) of combined data from all animals within the designated group. (TIF) [file ppat.1008339.s002.tif]

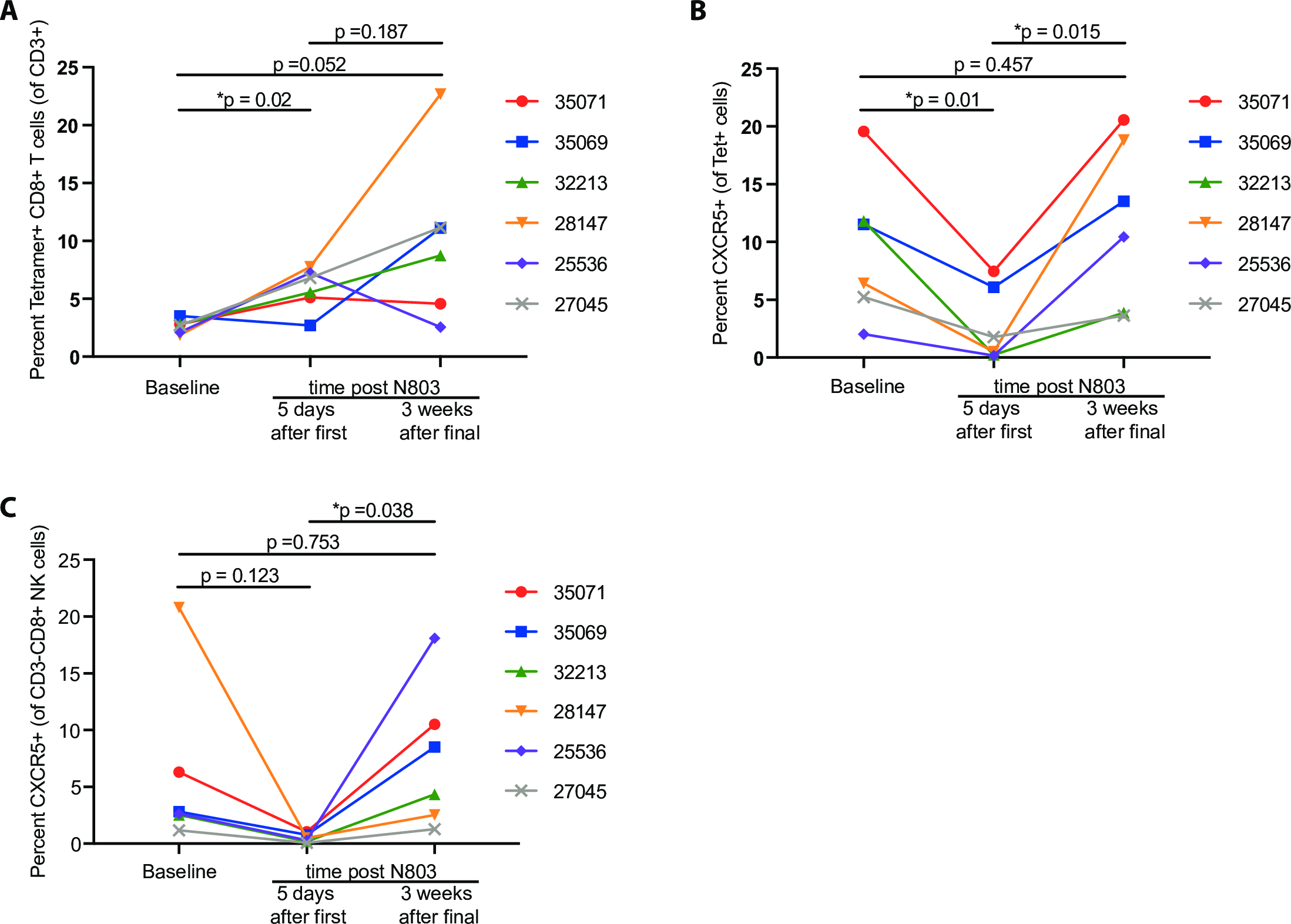

Supplement: S3 Fig — (A) Percent SHIV-specific CD8+ T cells as measured by MHC class I tetramer staining in lymph nodes prior to N-803, 5 days after N-803, and 3 weeks after the final N-803 administration. (B) CXCR5 staining on SHIV-specific CD8+ T cells in lymph nodes prior to N-803, 5 days after N-803, and 3 weeks after the final N-803 administration. (C) CXCR5 staining on NK cells in lymph nodes prior to N-803, 5 days after N-803, and 3 weeks after the final N-803 administration. P values were calculated using a paired t-test. *, P<0.05; **, P<0.01; ***, P<0.001. (TIF) [file ppat.1008339.s003.tif]

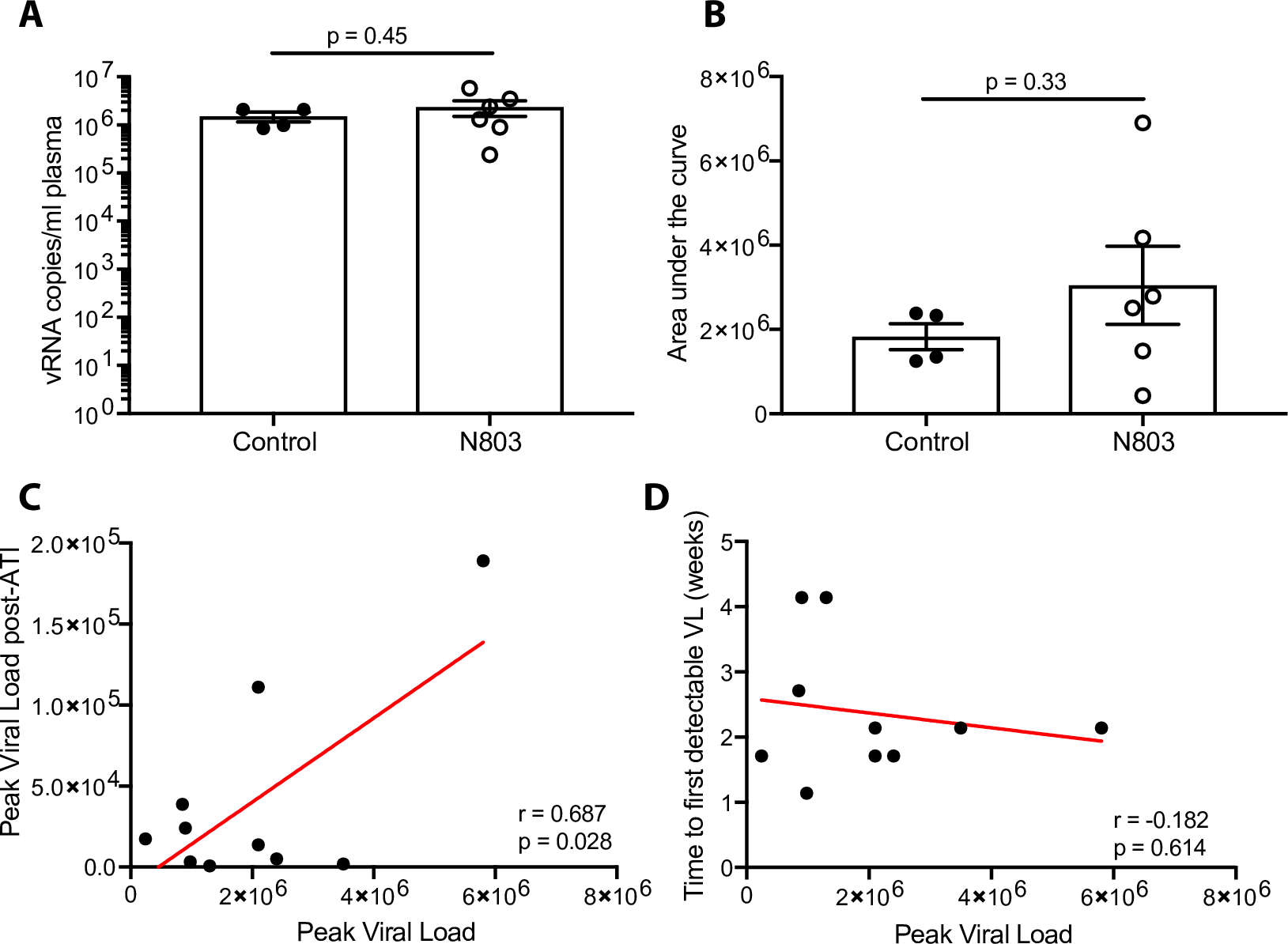

Supplement: S4 Fig — (A) Peak plasma viral loads and (B) area under the curve of viral loads prior to ART discontinuation. (C) Correlation of peak viral load post-ART release with pre-ART peak viral load. (D) Correlation of the time to the first detectable viral RNA in plasma after ART release with pre-ART peak viral load. Data shown are means (± SEM). P values were calculated using a Mann-Whitney test (A, B), and linear regression with Pearson’s correlation (C, D). *, P<0.05; **, P<0.01; ***, P<0.001. (TIF) [file ppat.1008339.s004.tif]

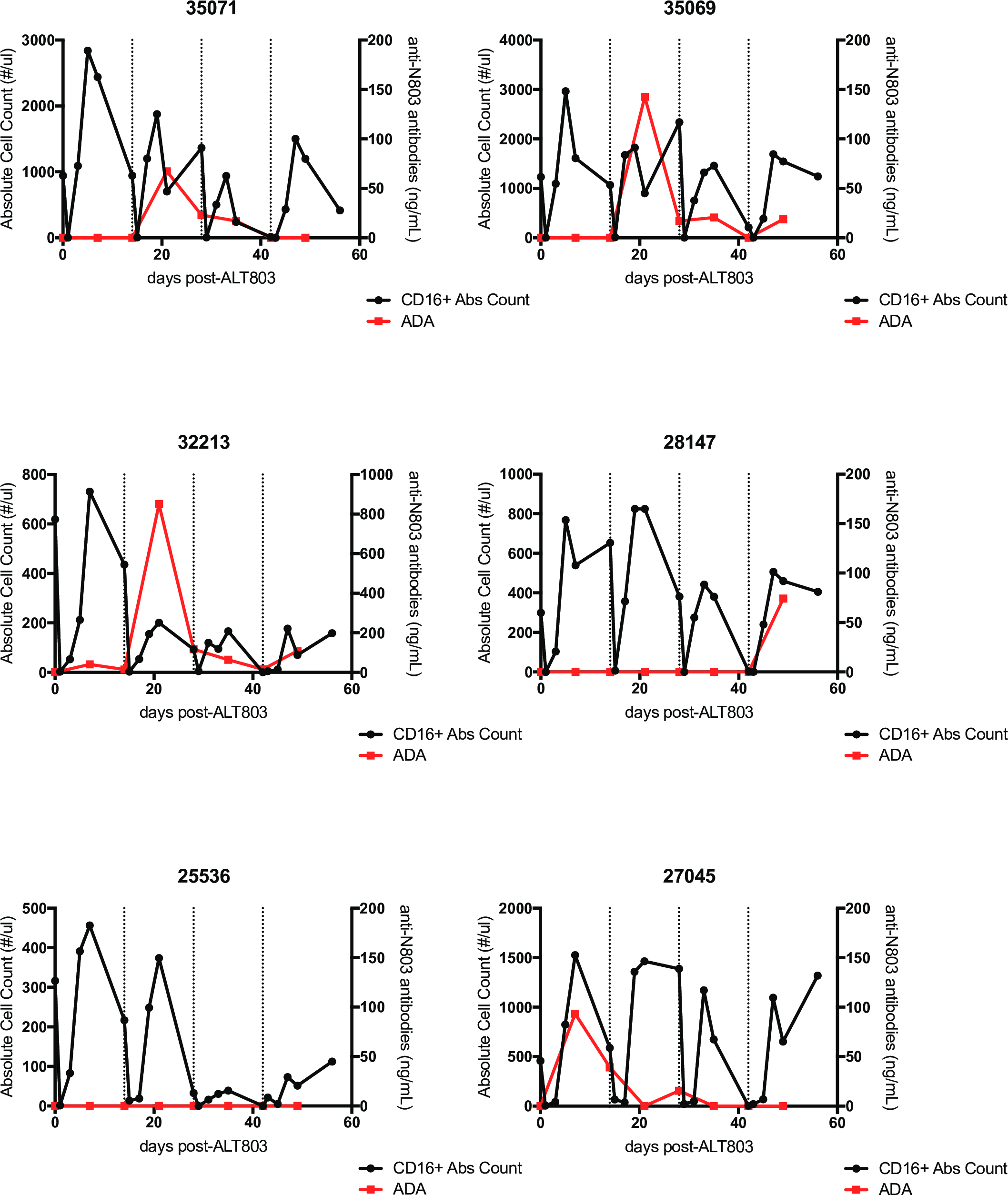

Supplement: S5 Fig — Anti-drug antibody development in each animal that received N-803 and the absolute cell count of CD16+ NK cells. Vertical dashed lines indicate times of N-803 administration. (TIF) [file ppat.1008339.s005.tif]

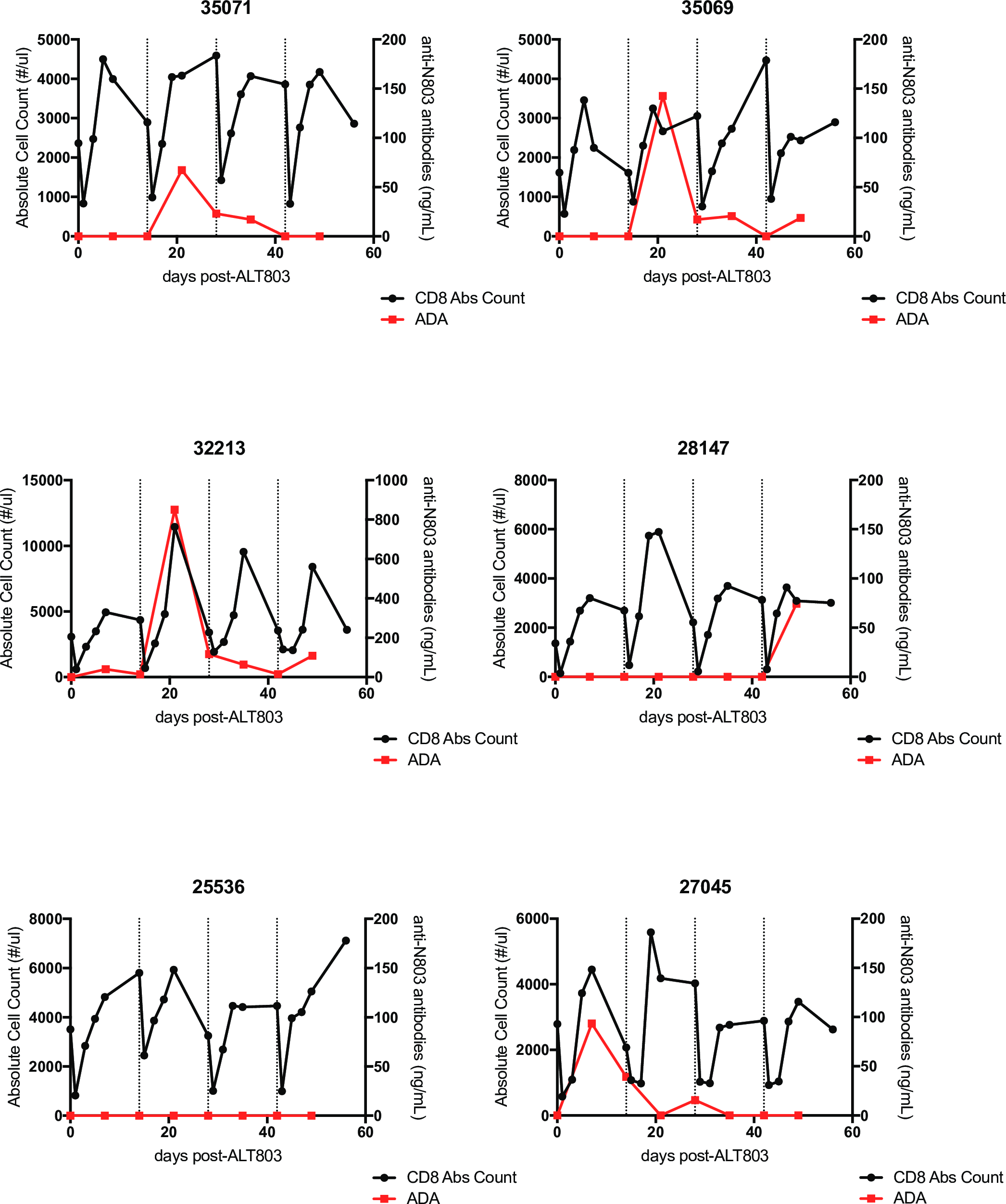

Supplement: S6 Fig — Anti-drug antibody development in each animal that received N-803 and the absolute cell count of CD8+ T cells. Vertical dashed lines indicate times of N-803 administration. (TIF) [file ppat.1008339.s006.tif]
